# Genome Editing for Earliness and Plant Architecture in Cucurbit Crops: Recent Advances and Future Perspectives

**DOI:** 10.3390/ijms27219697

**Published:** 2026-10-29

**Authors:** Lili Wan, Zhuanrong Wang, Jian Ren, Jiaqi Wei, Hongxia Zeng, Xiaoyong Li, Jiuhong Qu, Jianshun Xiong, Mi Tang

**Affiliations:** Institute of Crop, Wuhan Academy of Agricultural Sciences, Wuhan 430345, China; wanlili13226@163.com (L.W.);

**Keywords:** cucurbit crops, genome editing, earliness, plant architecture, CRISPR/Cas, precision breeding

## Abstract

Earliness and plant architecture are major breeding targets in cucurbit crops because they influence crop duration, early yield, planting density, canopy management, and production efficiency. This review summarizes genetic, molecular, and genome-editing advances related to flowering, sex expression, fruit development, stem elongation, branching, and canopy architecture in cucumber, melon, watermelon, Cucurbita species, and other cucurbits. Current evidence shows that earlier flowering or increased femaleness does not necessarily lead to earlier commercial maturity, while compact growth must be evaluated together with yield, fruit quality, and environmental adaptation. CRISPR/Cas9 knockout remains the most established editing approach, whereas base editing, cis-regulatory editing, multiplex editing, developmental-regulator-assisted regeneration, and DNA-free RNP delivery offer promising routes toward more precise trait optimization. Major constraints include genotype-dependent transformation, pleiotropic effects, limited validation in elite germplasm, and scarce direct evidence for shortening fruit-development duration. An evidence-guided framework is proposed that links trait definition, functional genomics, target prioritization, tailored genome editing, whole-plant evaluation, and breeding deployment. Future progress will depend on quantitatively tuned alleles and coordinated optimization of reproductive timing, canopy architecture, productivity, fruit quality, and environmental stability.

## 1. Introduction

Cucumber (*Cucumis sativus*), melon (*Cucumis melo*), watermelon (*Citrullus lanatus*), squashes and pumpkins (*Cucurbita* spp.), and other related cucurbits are cultivated under increasingly intensive production systems. These systems favor rapid crop turnover, predictable harvest periods, and high early marketable yield. Consequently, earliness has substantial economic importance in protected cultivation, early-season field production, and multiple-cropping systems. It is also an important breeding target for reducing generation intervals. Genetic mapping studies in cucumber and other cucurbits have identified numerous loci associated with flowering time, female flowering, and maturity. However, the phenotypic effects of these loci often vary across developmental stages and environmental conditions [1,2,3,4]. Plant architecture determines how efficiently cucurbit canopies occupy space and convert intercepted light into marketable yield. Key architectural traits include vine length, internode length, branching pattern, leaf and petiole angle, and shoot determinacy. These traits influence planting density, pruning and training requirements, canopy light distribution, and compatibility with labor-saving or mechanized production systems. Natural regulatory variation at *YABBY1*, together with genome-edited compact-canopy modules, demonstrates that canopy architecture can be deliberately modified without treating dwarfism as the primary breeding objective [5,6]. Earliness and plant architecture are developmentally interconnected. Their coordination involves shoot apical meristem fate, floral transition, sex determination, hormone signaling, branching, internode elongation, and source–sink relationships. Temperature and photoperiod can modify determinate growth through *TFL1*-related and cold-responsive pathways [7,8]. In addition, interactions between flowering–branching modules and architecture-related genes can produce coordinated multi-trait phenotypes. Therefore, the central breeding challenge is not simply to shorten the crop cycle or reduce plant size independently. Rather, developmental timing and canopy architecture must be co-optimized without compromising fertility, yield, fruit quality, or environmental stability.

Reproductive-transition earliness, as defined in this review, refers to the timing of floral transition and first flowering. These traits provide informative measures of reproductive development but do not directly indicate commercial maturity. Comparative analyses of PEBP family genes and flowering regulators in cucumber support a broadly conserved *FT/TFL1*-related regulatory framework [9,10]. At the same time, species-specific allelic variation can modify this framework. For example, variation in *CsPHYB* can advance flowering, indicating that photoreceptor signaling also contributes to reproductive-transition earliness [11]. Female-flowering earliness describes the timing and abundance of female reproductive sites. Relevant traits include days to the first female flower, node of the first female flower, female-flower frequency, and the capacity to set fruit soon after reproductive transition. These traits are particularly important in monoecious and gynoecious cucurbits because fruit production depends directly on the availability of female flowers. Sex-expression genetics should therefore be considered an integral component of reproductive earliness. However, gynoecy or a lower node of the first female flower does not, by itself, demonstrate earlier physiological or commercial maturity [12,13,14]. Fruit developmental earliness refers to the interval from anthesis or fruit set to commercial harvest. Physiological ripening represents a later developmental program and should be distinguished from this interval. Its biological meaning also differs between climacteric and non-climacteric cucurbits. Studies of rootstocks and production systems in watermelon further show that flowering time and fruit maturity can respond differently to cultivation conditions [15]. Accordingly, delayed ripening or extended shelf life should not be classified as early maturity solely because a fruit-development gene has been edited.

Stem and vine architecture encompasses total vine length, plant height, internode length, node-production dynamics, dwarfism, and compact growth. These traits determine the physical dimensions of the canopy and the spatial position of reproductive nodes. However, internode shortening does not necessarily alter the node number at which the first female flower appears. Comparative analyses among cucumber cultivars further indicate that node production and internode development change dynamically along the vine [16]. Thus, vine length should be interpreted as the integrated outcome of both node-production rate and internode elongation. A broader definition of plant architecture also includes determinacy, axillary-bud initiation and outgrowth, lateral-branch number, leaf size and shape, petiole and leaf angle, tendril identity, and other outputs of shoot meristem development. These components are not genetically independent. Regulators of tendril development and lateral-organ identity can also influence flowering, branching, and yield-related traits [17,18]. Such pleiotropic effects are important when evaluating architectural genes as breeding or genome-editing targets.

Genome editing provides a translational bridge between genetic discovery and crop improvement. It complements QTL mapping, GWAS, mutant analysis, and functional genomics by enabling the direct manipulation of candidate and functionally validated genes. In this review, the available evidence is organized in a stepwise framework that begins with phenotype definition and proceeds through genetic and molecular characterization, regulatory pathway analysis, gene validation, genome-editing evidence, edited phenotypes, breeding value, current limitations, and prospective editing strategies. This framework distinguishes three related but conceptually distinct levels of evidence: causal biological knowledge, technical editability, and agronomic deployability.

This review focuses on cucumber, melon, watermelon, Cucurbita species, and other Cucurbitaceae for which sufficiently strong genetic and functional evidence is available. Despite substantial progress in identifying genes associated with earliness and plant architecture, the extent to which these genes have been directly validated through genome editing remains uneven across traits and species. Moreover, many genes supported by genetic, molecular, or functional evidence have not yet been evaluated as genome-editing targets. The translational potential of these unedited candidates therefore remains insufficiently resolved. Given these knowledge gaps, the review evaluates the current evidence for genome-edited genes controlling earliness and plant architecture and identifies functionally supported genes that remain credible targets for future editing. It further examines editing strategies that may enable the coordinated improvement of early maturity and compact plant architecture. Notably, the central challenge is not simply to modify individual traits, but to determine whether developmental timing and canopy architecture can be co-optimized while maintaining fertility, yield, fruit quality, and environmental stability.

More broadly, the review assesses how advances in functional genomics and genome editing can translate expanding knowledge of earliness and plant architecture into precision breeding of cucurbit crops. Particular emphasis is placed on linking biological causality with editability and agronomic utility. To address these knowledge gaps, this narrative review draws primarily on published studies of genome editing, genetic and functional characterization, and developmental and agronomic traits in cucurbit crops. Genetic mapping and omics studies are also considered to explain how candidate genes have been identified, while studies in non-cucurbit species are used only to provide mechanistic or methodological context. Because the available evidence varies across genes, traits, and species, we classify it into three levels according to the strength and type of experimental support. Level I includes genes directly edited in cucurbit species with experimentally characterized effects on the trait of interest. Level II includes genes supported by strong genetic or functional evidence in cucurbits but without direct genome-editing validation of that trait. Level III includes candidate genes identified mainly through QTL mapping, genome-wide association studies (GWAS), transcriptomic analyses, comparative genomics, or orthology, for which causal or functional evidence remains limited. These levels are assigned separately for each trait, as genome-editing validation of a plant architecture phenotype does not necessarily demonstrate an effect on earliness. Furthermore, the strength of biological evidence is evaluated independently of potential breeding value, since a validated gene or edited allele may not always confer agronomically desirable traits.

## 2. Genome-Editing Platforms in Cucurbits

### 2.1. CRISPR/Cas9 and Transgene-Free Editing

CRISPR/Cas9 remains the most widely used genome-editing platform in cucurbits. Early studies demonstrated efficient gene knockout in watermelon [19] and the recovery of non-transgenic edited cucumber plants after segregation [20]. More recent work has focused on developmental traits with direct breeding relevance, including sex determination, internode elongation, canopy geometry, and flowering–branching modules [6,21,22,23,24]. The key question is therefore no longer simply whether mutations can be generated, but whether a specific edited allele can improve an agronomic trait in a breeding-relevant genetic background. In sexually propagated cucurbits, segregation of editing constructs can yield transgene-free progeny, as shown in cucumber [20,21]. The approach works when transformation and generation advancement are efficient, but it does not remove the genotype dependence and tissue-culture burden that regeneration itself carries.

### 2.2. Transformation and Regeneration

The recovery of edited plants, rather than guide-RNA design, remains a major practical constraint in most cucurbits. Conventional Agrobacterium-mediated transformation is strongly genotype dependent and can result in low regeneration efficiency, long tissue-culture cycles, chimerism, and somaclonal variation. An optimized transformation procedure enabled stable editing of *ERECTA* homologs in cucumber, melon, and squash and provided the first reported heritable genome editing in melon and squash [23]. Developmental regulators provide a complementary way to improve regeneration. In watermelon, *AtGRF5* increased transformation efficiency to approximately 24.7% and was combined with CRISPR editing [25]. In melon, *AtGRF5* and *AtPLT5* improved transformation in two genotypes and enabled recovery of *CmPDS-edited* plants from recalcitrant material [26]. These advances are important because genome editing must ultimately work in elite commercial germplasm, not only in transformation-friendly laboratory genotypes.

Editing efficiency measured in transformed tissue is not breeding efficiency. Chimeric shoots, poor germline transmission, and genotype-specific regeneration can stop apparently efficient editing systems from yielding stable elite lines. Platform comparisons should report regeneration rate, editing frequency, zygosity, chimerism, germline transmission, and recovery of transgene-free progeny as separate metrics. Genome structure also affects target design. Cucumber, melon and watermelon are commonly diploid crops with 2*n* = 14, 24 and 22 chromosomes, respectively [27,28,29]. Cultivated *Cucurbita pepo* generally has 2*n* = 40 chromosomes and retains signatures of an ancient whole-genome duplication [30]. Reference assembly sizes, estimates of genome size and ploidy must be distinguished. Functional target-copy number, paralog sequence similarity and genotype-specific sequence variation, rather than chromosome number alone, influence editability (Supplementary Table S1). Transformation, regeneration and editing efficiencies measure different outcomes. In a watermelon *AtGRF5*-assisted transformation system, a transformation efficiency of approximately 24.73% was reported in the tested background [25]; this is not the proportion of independently regenerated plants with stably inherited, transgene-free target edits. Comparisons should report explicit denominators and distinguish explants, transformed tissues, regenerated individuals, independent events, edited genotypes, chimeric plants and heritable progeny (Supplementary Table S2).

### 2.3. Multiplex Editing

A Pol II-driven sgRNA/Csy4 system increased editing efficiency in melon and watermelon and frequently generated large-fragment deletions between paired target sites [31]. Although these experiments did not target earliness or plant architecture, they provide a technical basis for future multi-locus or multi-element editing of developmental networks. For ideotype breeding, the value of multiplex editing lies in combining appropriately tuned alleles across partially independent pathways rather than simply stacking severe null mutations. A useful goal would be to combine earlier reproductive transition, moderate internode shortening, and reduced nonproductive branching. The number of edited loci is less important than the resulting whole-plant phenotype. The potential of multiplex editing is supported by the reported performance of the Pol II–Csy4 system, which achieved *PDS* editing efficiencies of 42.82% in melon and 41.48% in watermelon, compared with 25.20% using a Pol II–tRNA system in melon [31]. However, these results reflect editing efficiencies under specific experimental conditions and do not necessarily translate into stable inheritance, recovery of homozygous mutants, or improved breeding outcomes. Beyond technical efficiency, successful multiplex editing also depends on the careful selection of target genes. Although genes within the PEBP, WIP, ERECTA, and ACS/ACO families may share sequence similarities, they can have distinct functions in plant growth and development. Therefore, the functions, expression patterns, and potential interactions of individual genes should be evaluated before simultaneously editing multiple members of the same family. Such an approach would help identify suitable gene combinations for improving earliness and plant architecture while minimizing unintended effects.

### 2.4. Precision Editing

Cytosine base editing has been demonstrated in melon using CmeIF4E as the target, showing that defined nucleotide conversions can be generated without relying on double-strand-break repair [32]. This proof of concept is relevant to the future recreation of favorable natural alleles for quantitative developmental traits. More precise editing platforms should be described conservatively. Base editing is well established in several crops [33], and prime editing can generate precise sequence changes in cucumber, melon and pumpkin [34]. However, robust and routinely deployable prime-editing systems for cucurbit earliness or architecture genes have not yet been demonstrated. Adenine base editing and prime editing should therefore be regarded as emerging or future strategies for these traits until direct cucurbit evidence becomes available. The practical performance of precision editing in elite cucurbit genotypes also depends on editing windows, sequence context, delivery, regeneration, molecular validation and stable inheritance. DNA-free delivery can avoid stable foreign-DNA integration but does not by itself eliminate genetic mosaicism or the need for progeny confirmation. Claims of molecular precision must therefore be distinguished from reproducible recovery of suitable breeding material.

### 2.5. Regulatory and DNA-Free Editing

Regulatory editing is particularly attractive for dosage-sensitive developmental genes. The *YABBY1* Bush locus provides a direct cucurbit example: editing a conserved 5′UTR region produces graded changes in stem length rather than a single knockout phenotype [5]. This result illustrates why promoter, untranslated-region, enhancer, or allele-specific editing may be more suitable than complete gene disruption for quantitative ideotype design. Preassembled CRISPR/Cas ribonucleoproteins avoid stable foreign-DNA integration and have long been attractive for DNA-free plant editing [35]. In melon, particle bombardment of Cas9 RNPs into shoot apical meristems generated heritable *CmACO1* edits without conventional cell culture [36], a direct demonstration of a DNA-free in planta route in a cucurbit crop. Cas9-mediated knockout and conventional transformation are currently the most established cucurbit technologies. Developmental-regulator-assisted regeneration, cytosine base editing, improved multiplex systems, and in planta RNP delivery are promising but less broadly validated across genotypes. Routine prime editing, systematic promoter editing, and broadly genotype-independent tissue-culture-free editing remain future objectives. Maintaining these distinctions is important to avoid equating technical feasibility with breeding readiness. Representative platforms and their current development status are summarized in Table 1.

## 3. Genetic Basis of Earliness

### 3.1. Flowering and Meristem Regulation

Floral transition in cucurbits is controlled by homologs of well-known flowering and meristem regulators, although the strength of functional evidence differs among species. Comparative analysis of the PEBP family shows diversification in *FT/TFL1*-related expression and interaction patterns [10]. In cucumber, *TFL1*/*TFL1d* respond to temperature and photoperiod and inhibit determinate growth [7], while *CsTFL1* interacts with *CsNOT2a* to maintain indeterminate meristem activity [37]. These findings place PEBP-family genes at the intersection of flowering time and shoot determinacy rather than identifying a single universal ‘earliness gene.’ Some cucurbit loci also provide clear links between environmental signals and development. A 5.5 kb LTR-retrotransposon insertion in cucumber *CsPHYB* causes a long-hypocotyl, early-flowering phenotype [11], while a cold-shock protein induced at low temperature modulates determinate growth [8]. These results make light- and temperature-response pathways relevant to protected-cultivation breeding, but they also emphasize the need to test edited alleles across environments rather than relying on strong loss-of-function mutations. Meristem identity ties reproductive timing to architecture. *CsLFY* is needed for shoot-meristem maintenance and flower development and acts with *CsWUS* [38]; *CsCLV2* loss disturbs shoot-apical-meristem maintenance and determinacy [39]. In watermelon, *ClTFL1* and its GRF partners offer direct evidence that a meristem-related module can jointly govern flowering and lateral branching [24].

### 3.2. Sex Determination and Female Flowering

Sex determination is an important component of reproductive earliness because fruit production requires female reproductive organs. A WIP-family repressor contributes to androecy and the evolution of dioecy in cucurbits [40], and the andromonoecious sex-determination gene predates the divergence of Cucumis and Citrullus [41]. Direct editing of *WIP1* homologs in cucumber and watermelon, discussed in Section 4, further supports the conserved role of this pathway. Ethylene is the best-characterized hormonal pathway controlling sex determination in cucurbits [12,13]. In cucumber, CRISPR/Cas9 and complementary genetic analyses established *ACS1G* as the gene responsible for gynoecy at the F locus [42]. In watermelon, *CitACS4* regulates monoecy/andromonoecy through ethylene biosynthesis [43], whereas a separate partial-andromonoecy locus identified by BSA-seq and GWAS remains unresolved at the causal-gene level [44]. In *Cucurbita pepo*, the ethylene receptors *CpETR1A*, *CpETR1B*, and *CpETR2B* contribute to female-flower initiation and sex determination [45,46]. *CpACO1A* is involved in ethylene biosynthesis and female-flower development [47], while *CpMYB62* regulates the genetic network controlling the female floral transition [48].

Auxin also contributes to the sex-determination network. Direct deletion of cucumber *CsARF3* causes an androecious phenotype, whereas overexpression increases the number of female flowers, demonstrating an essential role in carpel development and sex determination [49]. *CsERF31* forms a positive feedback loop that promotes female-flower development [50], and expression differences associated with the F and M loci further support ethylene-mediated regulation [51]. In *C. pepo*, the jasmonate-biosynthesis gene *CpLOX3A* affects male and female flower opening and early fruit development through jasmonate–ethylene crosstalk [52]. Additional regulatory layers include temperature-associated DNA methylation changes in cucumber [53], epigenetic regulation in melon [54], and an miR164-resistant *CpCUC2B* mutation that alters carpel arrest and floral-boundary specification in *C. pepo* [55].

Together, these studies show that ethylene-centered regulation is broadly conserved across cucumber, melon, watermelon, and Cucurbita, but the specific genes, floral phenotypes, and breeding consequences differ among species [41,56,57]. Therefore, functional evidence from one cucurbit species should not be assumed to predict the effect of editing an orthologous gene in another species.

### 3.3. Female-Flower Position and Capacity

Female-flower timing, nodal position, and frequency decide when potential fruiting sites become available. QTL analysis has identified loci associated with early female flowering in zucchini [4], while genetic resources in cucumber include loci related to flower and fruit traits [58]. These female-flowering traits should be measured separately from days to first flower because the timing of male and female flowering can be genetically regulated differently. Female-flower earliness should also be distinguished from compact plant architecture. Short internodes reduce the physical height of each node but do not necessarily reduce the node number at which the first female flower appears. Therefore, a dwarf or compact plant may appear to bear fruit closer to the ground simply because its nodes are more closely spaced, rather than because female flowers develop earlier. When sex-expression alleles are combined with compact architecture, earlier female flowering should therefore be confirmed by measuring the node number of the first female flower and/or the time to female flowering, rather than inferred from plant height alone.

### 3.4. Fruit Set and Maturity

Early flowering does not necessarily lead to early yield because fruit set and subsequent fruit development are separate stages. Ethylene biosynthesis and signaling are involved in fruit set and early fruit development in zucchini squash [59]. Jasmonate also contributes to this developmental stage: mutation of *CpLOX3A* in *C. pepo* affects flower opening and promotes parthenocarpic fruit development, demonstrating crosstalk between jasmonate and ethylene during flower maturation and early fruit development [52]. In cucumber, SF1 regulates the stability of ACS2, thereby controlling ethylene dosage, cell division, and fruit elongation [60]. These findings demonstrate important hormonal control of fruit set and early fruit growth, but they do not show that fruits reach commercial maturity earlier.

The interval from flowering to harvest is influenced by several processes, including parthenocarpy, pollination and fertilization, ovary development, cell division, and cell expansion. Effects on any of these processes should therefore be distinguished from a direct reduction in fruit-development duration. Fruit ripening must also be considered separately. Ethylene plays a major role in the ripening of climacteric melon, whereas ripening is regulated differently in non-climacteric cucumber and watermelon [61]. Genome-editing studies should therefore distinguish among fruit set, early fruit growth, the duration from anthesis or fruit set to commercial harvest, physiological ripening, and shelf life. A change in one of these traits should not automatically be interpreted as earlier commercial maturity. Earliness endpoints should be reported separately: days to floral transition, node and days to first female flower, first successful fruit set, the interval from anthesis or fruit set to a crop-specific commercial harvest standard, days to first marketable harvest, and cumulative early marketable yield. Cucumber and summer squash are commonly harvested as immature fruits, whereas melon and watermelon have different harvest and quality criteria. Earlier flowering or increased femaleness should not be described as improved early marketable yield unless the latter was directly measured under a specified harvest window.

Comparisons with other crops provide hypotheses but not direct evidence of shorter cucurbit harvest intervals. In tomato, CRISPR disruption of *RIN* alters fruit ripening without establishing earlier commercial harvest, while natural *rin*, *nor* and *Cnr* alleles show interacting and allele-dependent ripening phenotypes [62,63]. *FW2.2*-related mechanisms principally concern fruit growth and size, which should not be equated with a reduction in anthesis-to-harvest duration. Existing cucurbit mechanisms involving *SF1–ACS2* and *CpLOX3A* provide initial functional leads [52,60]; causal effects on commercial developmental duration remain to be tested.

### 3.5. Mapping to Edit-Ready Targets

Earliness is a complex quantitative trait that is strongly influenced by environmental conditions. QTL studies in bitter gourd, citron watermelon, zucchini, cucumber, and other cucurbits have identified loci associated with flowering time, female-flower timing or position, fruit traits, and other earliness-related agronomic traits [2,3,4,58,64]. However, identifying a QTL or marker associated with a trait does not directly provide a suitable target for genome editing. Because many loci have relatively small effects, further fine mapping is needed to identify the causal gene or regulatory variant responsible for the observed phenotype. Potential editing targets should then be evaluated through functional validation, assessment of the direction and strength of the allele effect, analysis of possible pleiotropic effects, and consideration of the intended breeding objective. These steps are particularly important for Level III candidates identified mainly through QTL, GWAS, or omics studies, because editing an annotated gene located within a mapped region may not reproduce the phenotype associated with that region. At present, the strongest Level I evidence for earliness-related traits includes direct editing of the *ClTFL1*–*ClGRF* module and *ClPID* for flowering-related phenotypes, and *CsWIP1*, *ClWIP1*, *ACS1G*, and *CsARF3* for sex expression and female-flower development [17,21,22,24,42,49]. Direct editing of *ClAP1* also provides Level I evidence for its role in floral initiation, but knockout inhibits floral initiation and therefore does not represent an earliness-improving allele [17]. Similarly, direct editing of *CmACO1* provides Level I evidence for fruit-ripening modification, but the reported phenotype is delayed ripening and extended shelf life rather than earlier commercial maturity [36]. Other genes have strong functional evidence but do not yet provide direct genome-editing evidence for the focal earliness phenotype. These Level II targets include *CsTFL1/CsTFL1d*, *CsPHYB*, *CsLFY/CsWUS*, *CsCLV2* in the context of earliness, *CitACS4*, several ethylene receptors and ACO/ERF-related regulators, *CpMYB62*, *CpCUC2B*, *CpLOX3A*, and SF1/ACS2 [7,11,37,38,39,43,45,46,47,48,50,52,55,60]. Notably, *CsCLV2* has direct genome-editing evidence for plant architecture, but the reported edited phenotype does not establish earlier flowering or earlier commercial maturity [39]. Level III evidence includes unresolved QTLs and loci associated with flowering time, female-flower position, sex expression, and other earliness-related traits [2,3,4,44,58,64]. An important remaining gap is the genetic control of the period from fruit set to commercial harvest. Compared with flowering and sex determination, direct genome-editing evidence for shortening fruit-development duration and achieving earlier commercial maturity is still very limited in cucurbits.

## 4. Genome Editing for Earliness

### 4.1. Flowering and Meristem Editing

Direct genome-editing evidence for flowering time remains limited in cucurbits compared with the large number of genes and loci identified through genetic mapping. However, studies in watermelon have provided clear evidence that genome editing can be used to modify flowering time. CRISPR/Cas9 knockout of *ClTFL1* resulted in earlier flowering and fewer lateral branches and tendrils, while knockout of *ClGRF8* or *ClGRF12* in the same regulatory module also promoted earlier flowering and reduced lateral branching [24]. These findings provide Level I evidence that the same regulatory module can influence both flowering time and plant architecture. However, the simultaneous changes in several traits also indicate a risk of pleiotropic effects. Therefore, complete loss-of-function alleles of these genes may not always be suitable for breeding elite cultivars. A similar issue is seen with other developmental regulators in watermelon. CRISPR/Cas9 knockout of *ClPID* produced dwarf plants that flowered earlier but also developed abnormal floral organs and round, non-lobed leaves [17]. In the same study, editing *ClTEN* disrupted tendril development, whereas editing *ClAP1* inhibited floral initiation [17]. These results demonstrate that genes controlling lateral-organ development can be successfully edited, but they also show that strong gene disruption may cause undesirable changes in other organs or developmental processes. For breeding purposes, weaker alleles or regulatory and tissue-specific editing may therefore be more appropriate when the aim is to modify flowering time or plant architecture while minimizing unwanted developmental effects.

### 4.2. Sex Determination Editing

CRISPR/Cas9 disruption of *CsWIP1* produced non-transgenic gynoecious cucumber plants with substantially more female flowers [21]. This provides Level I evidence that genome editing can be used to modify sex expression and increase the number of female reproductive sites, which is also useful for hybrid seed production. However, producing more female flowers does not necessarily result in earlier commercial maturity. Earlier maturity can only be demonstrated if subsequent processes, such as fruit set and the time from anthesis to harvest, are also evaluated. In watermelon, CRISPR/Cas9 editing of *ClWIP1* similarly produced gynoecious plants [22], providing independent evidence for the conserved role of *WIP1* in cucurbit sex determination. As in cucumber, this finding demonstrates successful modification of sex expression but does not by itself demonstrate earlier fruit maturity. Two additional cucumber genes have Level I evidence for direct modification of sex expression. CRISPR/Cas9 and complementary genetic analyses established *ACS1G* as the gene responsible for gynoecy at the F locus [42]. Direct deletion of *CsARF3* causes androecy, whereas overexpression increases the number of female flowers [49]. These studies demonstrate direct manipulation of genes controlling female-flower development and sex expression. However, as with *WIP1*, changes in sex expression should not be interpreted as earlier commercial maturity unless fruit set, fruit-development duration, and harvest timing are also measured. Other components of the sex-determination network remain Level II for the breeding outcomes considered here. In watermelon, *CitACS4* has strong functional genetic evidence for the regulation of monoecy/andromonoecy but has not been directly validated through genome editing for the focal trait [43]. In *C. pepo*, *CpETR1A/CpETR1B/CpETR2B*, *CpACO1A*, and *CpMYB62* have functional evidence from mutant and genetic analyses [45,46,47,48], while *CsERF31* has strong functional evidence in cucumber [50]. *CpCUC2B* also affects carpel arrest and floral-boundary specification in *C. pepo* [55]. In contrast, the partial-andromonoecy locus identified in watermelon by BSA-seq and GWAS remains Level III because its causal gene has not yet been resolved [44]. Because many of these regulators participate in broader hormone-signaling and developmental pathways, future editing strategies should consider allele strength, tissue or developmental-stage specificity, and possible pleiotropic effects. The breeding value of edited sex-expression alleles should ultimately be assessed by their effects on fruit set, early yield, and commercial maturity rather than by female-flower number alone.

### 4.3. Fruit Development and Maturity Editing

DNA-free in planta RNP editing of *CmACO1* in melon reduced ethylene production and extended fruit shelf life [36]. This study demonstrates that heritable genome editing can be achieved through in planta RNP delivery and that this approach can be used to modify fruit ripening. However, the edited phenotype was delayed ripening and longer shelf life rather than earlier commercial maturity. Therefore, this study should not be considered direct evidence that genome editing can shorten the crop cycle. Compared with the progress made in editing flowering time and sex determination, direct genome editing to shorten the period from anthesis to commercial harvest remains limited in cucurbits. Future studies should identify and edit genes that specifically control the duration of fruit development, accurately measure the time from anthesis or fruit set to commercial maturity, and evaluate edited lines under different environmental conditions. Such studies will be necessary to determine whether earlier maturity is caused by the edited allele itself rather than by environmental stress or abnormal fruit development that may reduce fruit quality. A research strategy for fruit-development earliness should begin with crop- and market-specific commercial harvest criteria, followed by time-resolved phenotyping and combined mapping, transcriptomic and functional analyses. Candidate alleles should be validated before editing, with quantitative or regulatory changes considered when complete knockout risks disrupting fruit quality. Edited materials should be compared with appropriate controls for fruit set, anthesis-to-harvest duration, marketable size, quality, early marketable yield and total yield in representative environments. Candidate effects inferred only from ripening or fruit size must remain explicitly provisional.

### 4.4. Target Prioritization for Earliness

We prioritized genes and loci related to earliness using the three evidence levels defined in the Introduction (Table 2). Level I indicates direct genome-editing evidence for a relevant reproductive trait in cucurbits; Level II indicates strong functional evidence without direct editing validation of the specific earliness outcome; and Level III covers candidate loci identified mainly through genetic mapping. These levels describe the strength and relevance of the evidence, not whether an allele is ready for breeding. Level I entries include *ClTFL1*/*ClGRFs*, *ClPID*, *CsWIP1*, *ClWIP1*, *ACS1G*, and *CsARF3* [17,21,22,24,42,49]. However, their reported effects are not interchangeable: some influence flowering time, whereas others primarily affect sex expression or female-flower development. Two additional edited genes illustrate why the phenotype must be considered carefully. *ClAP1* knockout prevents floral initiation, confirming its role in flowering but not providing an allele that promotes earliness [17]. Editing *CmACO1* reduces ethylene production and delays fruit ripening, extending shelf life rather than advancing commercial maturity [36]. Level II includes genes with cucurbit-specific functional evidence related to flowering, meristem activity, sex determination, flower opening, or fruit development, but without direct editing evidence that establishes an improvement in the relevant earliness outcome. These include *CsTFL1*/*CsTFL1d*, *CsPHYB*, *CsLFY*/*CsWUS*, *CsCLV2*, *CitACS4*, *CpETR1A*/*CpETR1B*/*CpETR2B*, *CpACO1A*, *CpMYB62*, *CsERF31*, *CpCUC2B*, *CpLOX3A*, and *SF1*/*ACS2* [7,11,37,38,39,43,45,46,47,48,50,52,55,60]. For example, *CsCLV2* has been edited successfully, but the reported dwarf and determinate growth phenotype does not demonstrate earlier flowering or commercial maturity [39]. It is therefore classified as Level II specifically for earliness, even though it has direct editing evidence for plant architecture. Level III comprises mapped loci associated with flowering time, the timing or position of female flowers, sex expression, and maturity-related traits [2,3,4,44,58,64]. Before these loci can be considered defined editing targets, the causal genes or variants must be identified, their effects and direction confirmed, and their performance tested across genetic backgrounds and environments. Overall, a higher evidence level does not necessarily imply greater breeding value: a mapped locus could yield a favorable allele after validation, while a directly edited gene could produce undesirable effects or substantial pleiotropy.

Successful genome editing alone is therefore not sufficient for breeding application. Edited lines should be evaluated throughout the major stages of reproductive development, including floral transition, appearance of the first female flower, first fruit set, fruit developmental duration, commercial maturity, and early yield. Studies should also evaluate allele strength, stable inheritance, pleiotropic effects, and agronomic performance under production-relevant environments. Together, these measurements can determine whether an edited allele is not only effective at modifying the target trait but also useful for breeding. The principal earliness-related genes, their evidence levels, editing status, and breeding implications are summarized in Table 2. Studies in Table 2 report different outcomes rather than a single measure of earliness. Editing *ClTFL1*/*ClGRFs* affects both flowering and branching [24], while editing *CsWIP1* or *ClWIP1* changes sex expression [21,22]. *ClPID* knockout promotes earlier flowering but also causes marked developmental defects [17]. By contrast, *CmACO1* editing delays ripening and improves shelf life [36]. Thus, evidence that an edited gene changes one reproductive trait should not be taken as proof of earlier harvest or higher early yield. Table 2 identifies the observed phenotype and explains what remains unproven for breeding.

## 5. Genetic Regulation of Plant Architecture

### 5.1. Meristem Fate and Determinacy

Plant growth habit is strongly influenced by the activity and developmental fate of the shoot apical meristem. In cucumber, *CsTFL1* helps maintain shoot apical meristem activity and prevents determinate growth and terminal flower formation through its interaction with *CsNOT2a* [37]. A premature-stop allele of *CsTFL1* causes the determinate-2 phenotype and is also associated with earlier flowering and changes in branching [65]. In watermelon, the branchless gene Clbl encodes a *TFL1* protein that regulates the number of lateral branches [66]. Direct genome editing provides further evidence for the role of this gene family: editing the *ClTFL1*–*ClGRF* module affects both flowering time and lateral branching [24]. Together, these studies show that *TFL1*-related genes can influence both plant architecture and reproductive timing, making them important targets for coordinating growth habit, branching, and flowering in cucurbit breeding. Other genes involved in shoot meristem maintenance also have important effects on plant architecture. Loss of *CsCLV2* in cucumber causes dwarfism and premature termination of the shoot apical meristem [39]. *CsLFY* is also required for shoot meristem maintenance and flower development through its interaction with *CsWUS* [38]. Because these genes regulate fundamental processes in meristem development, strong disruption may affect several developmental traits at the same time. Therefore, the strength and spatial pattern of gene modification should be carefully considered when these genes are targeted for breeding. For breeding applications, TFL1-related genes are promising targets because they regulate meristem fate, flowering time, and branching. However, the effects of different alleles and the intended harvesting system should be considered when selecting targets. In contrast, disrupting *CLV2* or *LFY* may impair meristem maintenance and reproductive development. Therefore, weaker alleles or modifications to gene regulation may be preferable when the goal is to achieve determinate growth without premature meristem termination.

### 5.2. Stem Growth and Compactness

Gibberellin (GA) metabolism and signaling play major roles in controlling internode length in cucurbit crops. In cucumber, *CsERECTA* promotes stem elongation by regulating GA biosynthesis [67]. Mutation of ent-kaurenoic acid oxidase causes GA deficiency and dwarfism [68], while *CsIREH1* regulates plant height by affecting DELLA stability [69]. *CsDELLA1* acts as a negative regulator of GA-mediated growth [70]. Similar GA-related mechanisms have been identified in other cucurbits. Mutations in *Cldf/GA3ox* cause dwarfism in watermelon [71,72]. In squash, *CpDw* and *CmaGA2ox2* encode or are associated with GA2-oxidase activity that restricts internode elongation [73,74], while mutation of LacDWARF1 causes GA-deficient dwarfism in sponge gourd [75]. Together, these studies show that GA-related regulation of stem growth is widely conserved across cucurbit species. However, for breeding purposes, moderate reductions in internode length are likely to be more useful than severe GA-deficient dwarfism.

Brassinosteroid (BR) metabolism is another important regulator of compact growth. In cucumber, *CsCYP85A1* has been proposed as a candidate gene for the super compact-1 mutation [76]. Mutation of *CsDET2* causes systemic BR deficiency and produces a super-compact phenotype [77], while mutations in *CsDWF5* and *CsDWF7* disrupt sterol or BR biosynthesis and also result in compact growth [78,79]. Direct genome-editing evidence is available for another BR-related regulator. In watermelon, *ClDUF21* interacts with the BR-biosynthetic protein ClDWF1, and genome editing of *ClDUF21* and its cucumber homolog produces similar compact phenotypes [80]. These findings support a conserved role for BR-related pathways in the control of cucurbit plant stature.

Auxin transport and signaling also contribute to stem elongation. In watermelon, the dwarf gene *Cldw-1* encodes an ABCB auxin transporter [81], and another mutation involving an ABC transporter has also been associated with dwarfism [82]. In melon, *CmSi* encodes an *ERECTA*-like receptor kinase that regulates stem elongation through auxin signaling and interacts with *CmPIN2* [83]. In comparison, cucumber *CsERECTA* influences stem elongation through GA-related regulation [67]. These findings suggest that related receptor-kinase systems can contribute to stem-growth regulation through different hormone-related mechanisms in different cucurbit species.

Stem growth is also controlled by mechanisms beyond hormone metabolism and signaling. In cucumber, deletion of the cyclin-dependent kinase inhibitor *CsSMR1* reduces both internode cell number and cell size, resulting in dwarf and determinate growth [84]. Other genes have been identified as candidates but still require stronger functional evidence. The classic cucumber cp locus was fine-mapped to a 220 kb region containing a CKX homolog as a proposed candidate, although its causal role has not been functionally confirmed [85]. *CsCLAVATA1* has also been proposed as a candidate associated with a cucumber dwarf mutation [86]. In watermelon, *ClNAC100* positively regulates GA-related and expansin-associated growth and affects both plant height and fruit size [87], while additional dwarf-associated loci have been identified through fine mapping or bulked-segregant analysis [88,89]. In melon, bulked-segregant analysis identified a genomic region associated with short internodes [90]. These studies illustrate an important distinction for genome-editing research: genes identified within mapped regions should not be treated as validated editing targets until their causal roles have been confirmed.

Despite differences among species, compact growth in cucumber, watermelon, melon, squash/pumpkin, and Luffa is repeatedly associated with GA and BR metabolism, auxin transport and signaling, receptor kinases, cell-cycle regulation, and meristem-related processes. However, the specific causal alleles are often species dependent. Quantitative genetic analysis of internode length in winter squash [91] and high-density mapping of the dwarf-vine trait in pumpkin [92] further indicate that plant architecture can be influenced by both major genes and the broader polygenic background.

For breeding, the value of compact growth should be judged by plant performance under the intended production system rather than by plant height alone. Severe disruption of hormone pathways may produce very short plants but can also reduce vigor, leaf area, fertility, or fruit growth. In contrast, moderate internode shortening may allow higher planting density and reduce the labor required for training and canopy management. Therefore, the breeding objective should be to obtain an appropriate degree of compactness while maintaining normal growth and reproductive performance, rather than simply producing the strongest possible dwarf phenotype. Direct editing of *ERECTA*-family genes and the *YABBY1* 5′UTR provides higher-priority evidence for controllable stem architecture [5,23,67]. By contrast, severe GA- or BR-deficient dwarf phenotypes can reduce vigor and may be poor commercial candidates. Alleles that moderately shorten internodes while preserving reproductive performance should be prioritized over maximal dwarfism.

### 5.3. Branching and Axillary Growth

Branching in cucurbits is controlled by several interacting hormonal and environmental pathways. In cucumber, CsBRC1 suppresses axillary-bud outgrowth by directly repressing the auxin efflux carrier CsPIN3 [93]. The CsTIE1–CsAGL16 and CsAGL16–CsGRF1 regulatory modules also control lateral-bud outgrowth through ABA catabolism [94,95]. In addition, the CsPHYB–CsPIF4–CsBRC1 module connects light signaling with ABA-dependent regulation of branching [96]. These studies provide strong functional evidence for several regulatory pathways that could be targeted to reduce excessive vegetative branching. Direct CRISPR/Cas9 editing validates *CsTIE1*, *CsAGL16*, and *CsGRF1* for architectural phenotypes; *CsPIF4* has direct functional disruption evidence and an independently reported CRISPR knockout phenotype [94,95,96,97]. Other pathway components must be assessed individually, and Level I status does not establish improved commercial yield. From a breeding perspective, the aim should not be to eliminate lateral branches completely, but to reduce unnecessary vegetative growth while retaining productive branches that contribute to flowering and fruit yield.

Branching is also genetically regulated in other cucurbit crops. In watermelon, *ClLs* is involved in lateral-organ initiation [17], *Clbl/TFL1* controls lateral-branch number [66], and the *ClTFL1*–*ClGRF* module affects both branching and flowering [24]. In melon, *CmSVPc* is required for lateral-branch development [98], and QTL mapping has identified additional candidate loci associated with branch number [99]. A dwarf and branchless watermelon germplasm combining *Cldw-1* and *Clbl* further demonstrates that different architecture alleles can be combined in the same genetic background [100]. This example shows that combining genes for plant height and branching is already feasible through conventional allele pyramiding and provides a useful basis for future multiplex genome-editing strategies. Branching targets should be assessed individually for direct editing, expression pattern, environmental sensitivity and productive lateral-branch retention. *CsTIE1*, *CsAGL16* and *CsGRF1* have direct CRISPR evidence, but the direction of *CsGRF1* loss-of-function is increased rather than reduced branching [94,95]. Quantitative regulation, rather than complete suppression of laterals, is preferable when fruit production relies on side shoots.

### 5.4. Leaf and Canopy Architecture

Leaf angle, size, and shape are important components of canopy architecture because they influence how plants occupy space and intercept light. In cucumber, *CsABCB19* encodes an auxin transporter, and its mutation produces erect leaves and a more compact leaf architecture [101]. *CsIAGLU* also regulates leaf petiole angle through auxin homeostasis, and CRISPR/Cas9 knockout directly demonstrated its role in petiole-angle control [102]. However, *CsIAGLU* knockout increases petiole angle [102]. Therefore, if the breeding objective is to develop a compact canopy for dense planting, simply knocking out *CsIAGLU* would not be the preferred strategy. Instead, breeding or editing approaches would more likely aim to maintain or strengthen alleles associated with smaller petiole angles or to increase *CsIAGLU* activity. Other developmental regulators affect leaf size and shape. In cucumber, *WOX1* controls leaf vein patterning and leaf size [103], while transcriptomic analysis of the *LITTLELEAF* phenotype suggests the involvement of auxin- and cytokinin-related regulatory networks [104]. Mutation of *CsGME* produces small, wrinkled leaves [105], and *CsRAX* factors affect leaf size and fruiting ability through auxin glycosylation [106]. In watermelon, *ClLL1* regulates lobed-leaf development by modifying auxin distribution [107]. Transcriptome profiling in melon also suggests that phytohormone-related pathways are involved in controlling the angle between the leaf stalk and stem [108]. Together, these studies show that leaf architecture is controlled by several developmental and hormone-related pathways.

For breeding, however, a compact leaf or canopy phenotype should be evaluated under the planting density for which it is intended. CRISPR/Cas9 knockout of *CsHLS1* in cucumber reduces petiole angle, leaf size, and overall plant width [6]. The edited line maintained total yield at normal planting density and showed higher photosynthetic rates and increased early yield at one higher planting density, although its full-season yield performance under high-density conditions still requires further validation [6]. This study is therefore an important example of evaluating an edited architectural trait not only by its morphological phenotype, but also by its performance within a specific production system. These findings highlight the importance of selecting appropriate editing strategies based on gene-specific effects and evaluating canopy-related traits in terms of photosynthetic performance, fruit set, and marketable yield at the intended planting density [6,102].

### 5.5. Tendril and Lateral-Organ Development

Tendrils are characteristic lateral organs of cucurbits, and their development is closely connected with the development of branches, leaves, and flowers. In watermelon, studies of *ClLs* and CRISPR-edited *ClTEN*, *ClPID*, and *ClAP1* show that changes in these developmental regulators can affect tendrils together with other lateral organs [17]. In summer squash, a pleiotropic mutation in a tendril-related TCP gene is associated with multiple flowering and increased yield [18]. These findings show that tendril development cannot be considered independently from other reproductive and vegetative traits. This developmental connection is important when tendril-related genes are considered as breeding or genome-editing targets. Removing tendrils or other lateral structures may simplify the canopy, but strong disruption of lateral-organ development could also have undesirable effects on flowering or reproduction. Therefore, the breeding value of a tendril-related phenotype should be evaluated together with its effects on other organs and on yield. Where strong loss-of-function alleles cause undesirable effects, weaker or regulatory alleles may provide a better way to reduce unwanted vegetative structures while retaining beneficial reproductive traits. Tendril and lateral-organ regulators should be approached cautiously because severe *ClPID*- and *ClLs*-associated perturbations can also affect floral and leaf development [17]. Where feasible, weaker or spatially restricted alleles merit testing. Fruit set, fertility, productive branching and management savings—not tendril absence alone—should determine breeding priority.

### 5.6. Hormonal Integration

Several hormone pathways contribute to the regulation of cucurbit plant architecture, but the strength of evidence differs among them. Gibberellin (GA) and brassinosteroid (BR) are the best-supported hormonal pathways controlling internode elongation and dwarfism. Natural, induced, and genome-edited alleles affecting these pathways have been reported across several cucurbit genera [67,71,72,73,74,77,80]. The repeated involvement of GA and BR across species suggests that these pathways have conserved roles in stem-growth regulation. However, many GA- or BR-deficient mutants show severe dwarfism, indicating that complete loss of gene function may not always produce a phenotype suitable for breeding.

Auxin, ABA, and light signaling also contribute to the regulation of branching and leaf architecture. In cucumber, regulatory modules such as *CsBRC1*–*CsPIN3* and CsPHYB–*CsPIF4*–*CsBRC1* connect auxin transport, ABA-related regulation, and light signaling with axillary-bud growth [93,96]. *CsPIF4* also promotes hypocotyl elongation in response to high temperature [97]. Because some of these pathways respond strongly to environmental conditions, edited phenotypes should be evaluated across relevant environments rather than under a single growth condition. Other hormonal pathways may also contribute to cucurbit architecture, but the current evidence is less complete. Cytokinin-related candidates and ethylene-mediated reproductive pathways may influence architectural traits, although their cucurbit-specific evidence is currently weaker than that for GA, BR, or auxin. Strigolactone is an important regulator of branching in other plant species, but current evidence in cucurbits is not sufficient to consider it an established genome-editing pathway for combined earliness and architecture breeding. Until stronger cucurbit-specific causal evidence becomes available, these less well-supported pathways should be presented as possible mechanisms or future editing targets rather than as validated breeding targets.GA, BR and auxin pathways are supported as major architectural regulators, but perturbation of central components may affect many organs. The CsTIE1–CsAGL16 ABA-related pathway also connects branching and drought responses [94,95], illustrating a potential architecture–stress trade-off. Edited genotypes should be compared for internodes, reproductive sites, marketable productivity and relevant environmental responses before hormone-pathway alleles are advanced.

## 6. Genome Editing for Plant Architecture

### 6.1. ERECTA Editing and Compact Architecture

*ERECTA*-family genes are among the best-supported genome-editing targets for compact plant architecture in cucurbits. Using an optimized transformation system, targeted editing of *ERECTA* homologs produced stable plants with shorter internodes and more compact growth in cucumber, melon, and squash [23]. The similar phenotypes observed across these species support a conserved role of *ERECTA*-family genes in stem growth and show that the same editing strategy can be applied to several major cucurbit crops. Further evidence comes from cucumber *CsERECTA*. Map-based cloning followed by CRISPR validation showed that *CsERECTA* knockout reduces endogenous GA levels and stem elongation, while application of exogenous GA3 partially restores the dwarf phenotype [67]. *CsERECTA* therefore represents a Level I architecture target supported by both direct genome editing and evidence for its role in GA-related stem growth. Shorter internodes and a smaller canopy may be useful for dense planting and could reduce the labor required for crop training and management. However, the degree of growth reduction is important. Complete loss of *ERECTA* function may produce stronger compactness than is desirable for commercial cultivars. Therefore, regulatory or partial-loss-of-function alleles should be compared with complete knockouts to identify an appropriate degree of compactness in elite breeding backgrounds.

### 6.2. YABBY1 Regulatory Editing

The *YABBY1* Bush locus provides a clear example of using regulatory editing to produce different degrees of compact growth. A conserved B-region in the *YABBY1* 5′UTR regulates translation efficiency. Deletion of this region increases *YABBY1* protein accumulation and suppresses stem elongation [5]. CRISPR/Cas9 editing of the *YABBY1* 5′UTR produced shortened-stem phenotypes in pumpkin, cucumber, and watermelon [5]. Importantly, this approach generated a series of alleles with different effects rather than a single knockout phenotype. *YABBY1* therefore provides Level I evidence that cis-regulatory editing can be used to modify a quantitative architecture trait in cucurbits. This example also has broader value for genome-editing-based breeding. For developmental genes whose effects depend on gene dosage, editing regulatory regions may allow the strength of the phenotype to be adjusted without completely disrupting gene function. Different alleles can then be evaluated to identify the degree of compactness that best fits planting density, fruiting habit, and the canopy requirements of individual cucurbit crops.

### 6.3. Hormone-Linked Compactness

Genome editing has also directly validated genes associated with hormone-regulated compact growth. In watermelon, CRISPR/Cas9 disruption of *ClDUF21* causes pronounced dwarfism, and the interaction between *ClDUF21* and the BR-biosynthetic protein ClDWF1 links this phenotype to BR homeostasis [80]. Editing the cucumber homolog *CsDUF21* similarly shortens internodes [80]. These results provide Level I evidence for a conserved role of DUF21 in compact growth across cucurbit species. However, severe dwarfism may also reduce plant vigor or reproductive performance and therefore requires careful evaluation before breeding use. Mutations that strongly disrupt GA- and BR-related genes can also produce dwarf or super-compact plants [68,71,72,73,77,79]. Although these studies clearly demonstrate the importance of GA and BR pathways in plant stature, severe hormone-deficient phenotypes may not be suitable for commercial production. When the breeding objective is moderate internode shortening rather than extreme dwarfism, approaches such as base editing, promoter editing, or recreation of favorable natural alleles may be more appropriate than complete loss of gene function.

### 6.4. Meristem and Canopy Editing

Genome editing of meristem-related genes can also modify plant architecture. Loss of *CsCLV2* function in cucumber causes dwarf and determinate growth, and genome-edited mutants reproduce this phenotype [39]. *CsCLV2* can therefore be classified as a Level I architecture target. However, premature termination of the shoot apical meristem may have broad developmental effects because determinate growth changes both the duration and spatial pattern of reproductive development. This pleiotropy must be considered when *CsCLV2* alleles are evaluated for breeding. Canopy architecture can also be modified directly through genome editing. CRISPR/Cas9 disruption of *CsHLS1* reduces leaf petiole angle, leaf size, and overall plant width, while functional analysis identifies *CsHLS1* as part of the *CsHLS1*–*CsSCL28* regulatory module [6]. The edited line maintained yield at normal planting density and showed improved photosynthetic performance and increased early yield at one higher planting density, although its full-season yield under high-density conditions still requires further validation [6]. This study is particularly useful because the edited phenotype was evaluated not only at the level of plant morphology but also in relation to planting density and crop performance.

### 6.5. Branching and Lateral-Organ Editing

Some genome-edited genes affect more than one component of plant architecture. In watermelon, editing the *ClTFL1*–*ClGRF* module changes both flowering time and lateral branching [24]. This makes the module relevant to both earliness and plant architecture and shows that genome editing can modify these two traits together. However, strong loss-of-function alleles may reduce branching more than is desirable and could affect reproductive capacity. The appropriate degree of branch reduction therefore needs to be determined for breeding applications.

Editing of other lateral-organ regulators in watermelon further illustrates the limitations of complete gene knockout. *ClPID* editing causes dwarfism and earlier flowering but also produces abnormalities in floral organs and leaves, while disruption of *ClLs* can eliminate branches, tendrils, and flowers [17]. These experiments provide important Level I functional evidence, but the resulting phenotypes may not be directly suitable for breeding. They also show that successful genome editing alone does not establish breeding value; the effects of an edited allele on plant growth, reproduction, and other agronomic traits must also be evaluated.

### 6.6. Evidence and Breeding Readiness

For plant architecture, Level I evidence refers to genes or regulatory regions that have been directly edited in cucurbits with a reported architectural phenotype. However, direct editing does not necessarily mean that the resulting allele is suitable for breeding. Current Level I targets include *ERECTA*-family genes, the *YABBY1* cis-regulatory region, *CsHLS1*, *CsCLV2*, *ClDUF21/CsDUF21*, the *ClTFL1–ClGRF* module, *ClPID/ClLs/ClTEN*, *CsIAGLU*, *CsIREH1*, *CsTIE1*, *CsAGL16/CsGRF1*, *CsPIF4*, *ClNAC100*, *CsRAXs*, and *ClLMI1* [5,6,17,23,24,39,67,69,80,87,94,95,96,102,106,107]. The *YABBY1* example is particularly informative because editing its regulatory region generated alleles with different effects rather than a single knockout phenotype [5]. In contrast, strongly pleiotropic edited mutants such as *ClPID* and *ClNAC100* show that direct functional validation does not necessarily produce an allele that is immediately useful for breeding [17,87]. Level II includes genes with strong functional evidence in cucurbits but without direct genome-editing evidence for the architectural trait considered here. Representative examples include *CsTFL1*, *Clbl*, *CsBRC1–CsPIN3*, *CsABCB19*, *CsDELLA1*, *CmSVPc*, *CsWOX1*, *CsGME*, and several GA- and BR-related dwarf genes [37,65,66,68,70,71,72,73,74,75,76,77,78,79,81,82,83,84,93,98,101,103,105]. These genes provide well-supported biological targets, but direct editing and evaluation of the desired architectural phenotype are still needed before they can be considered Level I targets. Level III includes unresolved candidate genes, QTLs, and regulatory pathways associated with plant height, branching, internode length, leaf size, or canopy architecture through mapping or omics studies [76,85,86,88,89,90,91,92,99,104,108,109]. These targets require further resolution of the causal gene or variant and validation of its phenotypic effect before specific genome-editing strategies can be designed. Importantly, evidence level and breeding value should be considered separately. Moving a target toward practical breeding requires not only successful editing but also selection of an appropriate allele strength, confirmation of stable inheritance, and direct evaluation of yield, fertility, plant vigor, and management benefits under relevant production systems. Table 3 summarizes the major plant-architecture targets, their evidence levels, editing status, and potential breeding value or limitations. Importantly, similar compact phenotypes may arise through distinct mechanisms, with different implications for breeding. For example, *ERECTA*-family editing affects internode elongation [23,67], *YABBY1* regulatory editing enables graded changes in stem growth [5], whereas CsCLV2 loss leads to premature meristem termination [39]. Thus, breeding value should be assessed not by reduced plant size alone, but by the underlying mechanism, allele effects, and potential trade-offs in plant vigor and reproductive productivity. Supplementary Table S2 provides reported editing outcomes, available quantitative phenotypic data, and evidence limitations to support this assessment.

## 7. Integrating Earliness and Plant Architecture

Earliness and plant architecture are closely connected through meristem fate, branching, internode elongation, leaf architecture, and reproductive development. However, modifying one of these traits does not necessarily improve the other. For example, the switch between determinate and indeterminate growth changes the duration of vegetative development and can concentrate reproduction into a shorter period, but it does not automatically lead to earlier marketable yield. *CsTFL1* alleles link meristem termination with flowering and branching [37,65], while *Clbl/TFL1* and the edited *ClTFL1*–*ClGRF* module similarly regulate branch number in watermelon [24,66]. These findings identify meristem fate as an important connection between earliness and architecture, but the effects on harvest earliness still need to be measured directly.

Hormonal pathways provide additional links between reproductive timing and plant architecture. Auxin contributes to several components of this network, including *CsARF3* in sex determination [49], *CsBRC1*–*CsPIN3* in branching [93], *CsABCB19* and *CsIAGLU* in leaf architecture [101,102], and *ClPID/ClLs-related* lateral-organ development in watermelon [17]. This broad involvement makes auxin signaling a promising target for coordinated trait improvement, but strong disruption of central auxin transport or response components may also cause substantial pleiotropic effects. GA and BR mainly regulate internode elongation and plant vigor, whereas ethylene has major roles in sex expression, fruit set, and ripening. Together, these hormone pathways connect canopy development with the timing and strength of reproductive sinks [42,56,60,77,80]. These connections also create important trade-offs. Reduced vegetative growth should not be assumed to redirect resources automatically toward earlier yield. Faster flowering may shorten the vegetative phase but can also reduce the source capacity required for subsequent fruit growth. The *ClTFL1/ClGRF* and *ClPID* editing phenotypes illustrate this issue, as early flowering was accompanied by substantial changes in branching, plant stature, or organ morphology [17,24]. Similarly, reducing nonproductive branches can lower pruning requirements and improve light penetration, but excessive suppression of branching may remove reproductive sites in genotypes that bear fruit on lateral vines. Branching alleles should therefore be evaluated according to crop species, fruiting habit, and harvest system rather than branch number alone. Severe GA- or BR-related mutations can generate strong compactness, but often at the cost of plant vigor, organ size, or reproductive performance. For breeding purposes, moderate compactness is therefore likely to be more useful than extreme dwarfism.

**Figure 1:**
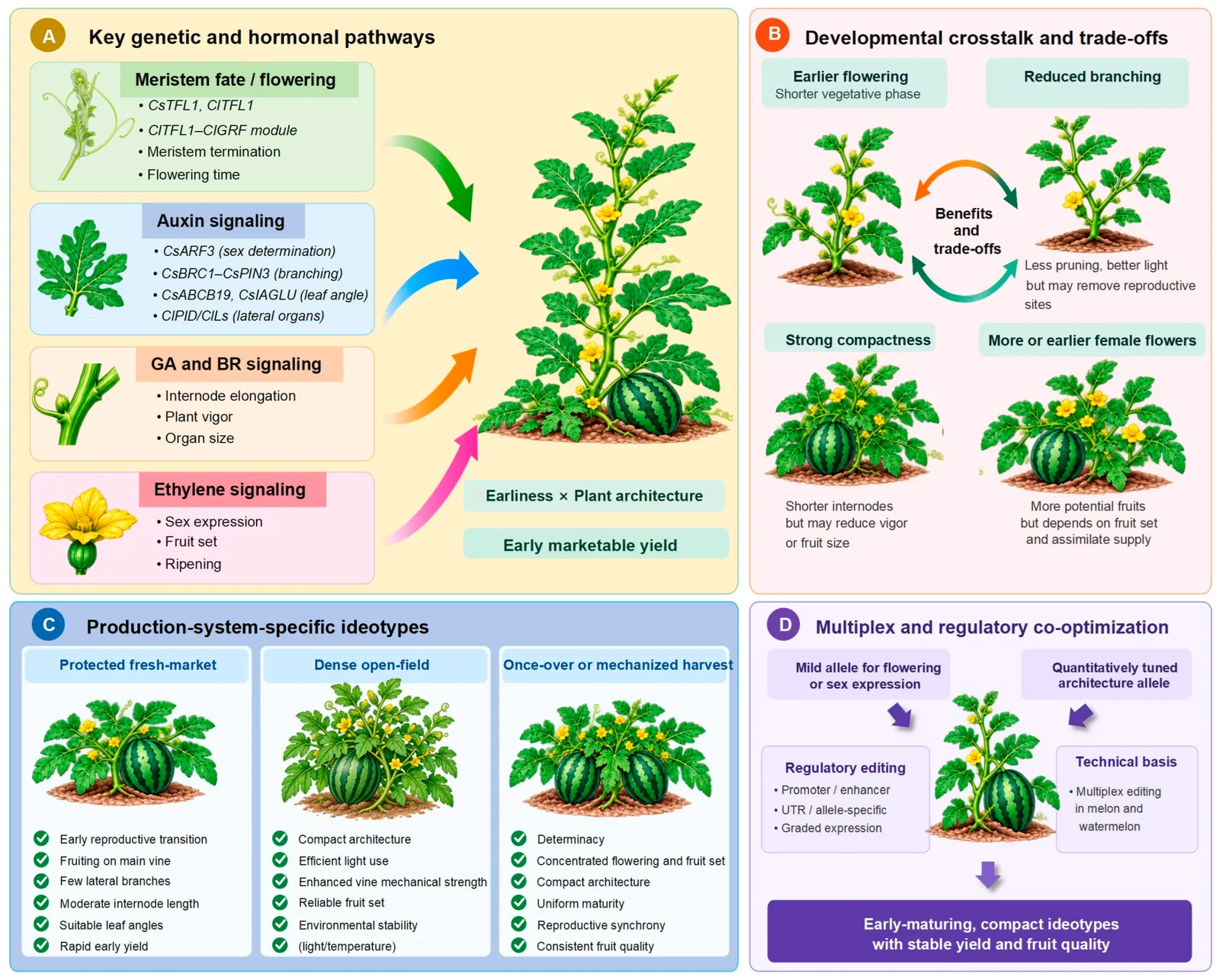
Integrated framework for co-optimizing earliness and plant architecture in cucurbits.

The same principle applies to reproductive traits. Increasing the number or earliness of female flowers provides more potential sites for fruit production, but early marketable yield ultimately depends on successful fruit set and sufficient assimilate supply. *CsHLS1-edited* plants provide an example in which a compact canopy was evaluated together with photosynthesis and yield across planting densities [6], while female-flower regulators can increase the availability of female reproductive sites [21,50]. Combining these traits should therefore be assessed at the whole-plant level, with particular attention to source–sink balance, rather than evaluating architecture or reproductive development separately. Figure 1 summarizes these major cross-trait relationships. On this basis, the desired combination of earliness and architecture should be defined according to the production system. In protected fresh-market production, a useful ideotype would combine a reasonably early reproductive transition, strong but manageable femaleness, fruiting on the main vine, few unnecessary lateral branches, moderate internode shortening, and leaf angles that maintain favorable light distribution at high planting density. The breeding goal is rapid early yield with reduced training and pruning labor, rather than maximal dwarfism. In dense open-field production, compact architecture should additionally support efficient canopy light use, lodging resistance, reliable fruit set, and stable performance across environments. Environment-responsive pathways require particular caution in this context. For example, phenotypes controlled by *CsPHYB*- and *CsPIF4-related* modules may vary with light and temperature conditions [11,96,97]. For once-over or mechanized harvest, stronger determinacy, concentrated flowering and fruit set, compact architecture, and uniform maturity become more important than continuous fruit production. Such systems may therefore benefit from stronger modification of meristem fate, although reproductive synchrony, yield, and fruit-quality uniformity must be demonstrated directly.

Gene-editing strategies for these ideotypes should therefore focus on coordinated but moderate modification of multiple traits. The *ClTFL1*–*ClGRF* module shows that a single editing target can influence both flowering and branching [24], whereas *YABBY1* cis-regulatory editing demonstrates that architectural traits can be adjusted quantitatively rather than through complete loss of gene function [5]. These examples support a rational multiplex strategy in which a mild allele affecting flowering or sex expression is combined with a quantitatively tuned architecture allele, instead of stacking several severe knockout mutations. Multi-guide editing systems already established in melon and watermelon provide a technical basis for this approach [31].

Comparison of the available studies further highlights the distinction between developmental effects and breeding benefits. Editing of the ClTFL1–ClGRF module affects both flowering and branching, whereas *ClPID* disruption causes additional abnormalities in plant stature and floral-organ development [17,24]. By contrast, YABBY1 cis-regulatory editing provides evidence for graded modification of stem growth, and CsHLS1 editing has been evaluated for canopy architecture and yield under different planting densities [5,6]. These studies differ in their target traits, experimental endpoints, and extent of agronomic validation, limiting direct comparisons of breeding value. Moreover, pleiotropic effects observed after editing a single regulator do not establish the performance of independently combined alleles. Such combinations require direct assessment of fruit set, productive branching, marketable yield, fruit quality, and environmental stability in the intended genetic backgrounds and production systems.

Allele strength will be especially important when targeting dosage-sensitive developmental regulators. Editing promoters, enhancers, untranslated regions, or specific alleles may generate graded changes in gene expression while preserving essential gene functions and reducing unwanted pleiotropic effects. *YABBY1* provides a direct precedent for this strategy in cucurbits [5]. Similar regulatory editing approaches could be explored for *TFL1*/*CEN*, *BRC1*, hormone biosynthesis and signaling genes, and sex-determination regulators with restricted expression domains. Overall, integrating earliness with plant architecture should aim to balance reproductive timing, canopy structure, source capacity, and fruit set rather than maximize any single trait. Combining appropriately tuned alleles and evaluating them under the target production system will be important for developing compact cucurbit ideotypes that achieve earlier marketable yield without compromising plant vigor, reproductive performance, or fruit quality. Figure 1 integrates these concepts into a unified framework linking the underlying genetic and hormonal pathways, developmental crosstalk and trade-offs, production-system-specific ideotypes, and multiplex/regulatory strategies for the coordinated optimization of earliness and plant architecture.

(A) Key genetic and hormonal pathways connecting reproductive development and plant architecture.
(B) Major developmental crosstalk and trade-offs between earliness and plant architecture.
(C) Production-system-specific ideotypes for protected fresh-market, dense open-field, and once-over or mechanized harvest systems.
(D) A strategy for multiplex and regulatory co-optimization using mild alleles and quantitative regulation to integrate earliness and plant architecture.

The genetic and hormonal relationships summarized in this figure are based on published studies with differing levels of experimental support. The proposed ideotypes and combinations of edited alleles represent prospective breeding applications rather than experimentally validated outcomes of simultaneous multi-trait editing. Direct validation of combined phenotypes and agronomic performance remains necessary.

## 8. Current Bottlenecks and Challenges

Transformation and plant regeneration remain the most immediate technical bottlenecks for genome editing in cucurbit crops. High editing efficiency at the cellular level has limited practical value if elite genotypes cannot be efficiently regenerated into fertile plants. Conventional tissue-culture-based approaches are often strongly genotype dependent and may require long regeneration periods. They can also result in chimerism, somaclonal variation, and incomplete transmission of edited alleles through the germline. Recent advances are beginning to address these limitations. Optimized transformation protocols applicable across several cucurbit crops [23] and developmental-regulator-assisted transformation systems developed in watermelon and melon [25,26] have improved transformation and regeneration efficiency. In parallel, segregation-based removal of transgenes and DNA-free in planta delivery of CRISPR ribonucleoproteins (RNPs) provide alternative routes for obtaining stable, transgene-free edited plants [21,36]. However, these approaches still need to be systematically evaluated in a broader range of elite germplasm. Comparisons should use standardized criteria, including regeneration efficiency, editing frequency, zygosity, chimerism, germline transmission, and recovery of edited progeny.

A second major limitation is the current dependence on indel-mediated loss-of-function mutations. Gene knockout is effective when complete loss of function produces a desirable phenotype, as in some negative regulators such as *WIP1*. However, complete knockout may be less suitable for dosage-sensitive genes involved in meristem development, hormone signaling, and plant architecture because strong mutations can also cause undesirable developmental effects. More precise editing strategies therefore need further development. Cytosine base editing in melon has demonstrated the feasibility of defined nucleotide conversion [32], while improved multi-guide systems have expanded the capacity for paired and multiplex editing [31]. Nevertheless, precise allele replacement, routine prime editing, and predictable engineering of multiple loci remain considerably less mature than conventional Cas9-mediated knockout. As genome editing moves toward breeding applications, both off-target mutations and unintended structural changes at the target site should be evaluated through careful guide design, molecular genotyping, segregation or backcrossing when appropriate, and phenotypic validation. Successful sequence modification alone is not sufficient evidence of a breeding-ready allele.

Biological complexity presents an equally important challenge. Genes controlling earliness and plant architecture often function in central developmental pathways, so improvement in one trait can negatively affect another. Accelerated flowering may reduce vegetative biomass, suppression of branching may remove potential fruiting sites, and severe disruption of gibberellin (GA) or brassinosteroid (BR) pathways may reduce plant vigor. Sex-expression phenotypes can also be influenced by temperature and photoperiod. The pleiotropic phenotypes produced by editing *ClTFL1/ClGRFs* and *ClPID* illustrate these trade-offs directly [17,24]. Promising edited alleles should therefore be tested under protected and open-field conditions, at different planting densities, and under contrasting temperature and light regimes. *CsPHYB*- and *CsPIF4-related* responses further illustrate the environmental sensitivity of flowering and growth pathways [11,96,97]. UV-A treatment can induce a robust dwarf phenotype in cucumber without reducing fruit yield [110], whereas waterlogging-induced adventitious rooting is regulated through ethylene–auxin–ROS signaling [111]. These studies demonstrate environmental plasticity and stress–hormone interactions, but neither provides evidence of earlier maturity. Comprehensive evaluation should include dynamic measurements of vine elongation, node production, branching, leaf angle, flowering, female-flower formation, fruit set, fruit growth, maturity, and early yield. AI-assisted phenotyping is beginning to emerge in cucumber, although current tools such as CucumberAI focus on automated fruit-morphology measurements rather than full developmental trajectories [112].

Finally, proof-of-concept mutants must be converted into agronomically useful elite breeding lines. A compact or determinate phenotype observed in a greenhouse has limited breeding value unless early and total yield, fruit quality, fertility, stress response, canopy-management requirements, and harvest efficiency are maintained or improved. Conventional Cas9-mediated knockout is currently the most established genome-editing strategy in cucurbits. Developmental-regulator-assisted transformation, cytosine base editing, improved multiplex systems, *YABBY1*-type cis-regulatory engineering, and in planta RNP delivery are emerging approaches or proof-of-concept technologies, depending on the crop and genotype. In contrast, routine prime editing, broadly genotype-independent tissue-culture-free editing, and predictive engineering of regulatory networks remain longer-term goals. The key translational challenge is therefore not simply to increase editing efficiency, but to combine efficient editing with stable inheritance, favorable multi-trait agronomic performance, and reliable deployment in elite cucurbit germplasm. Regulation and market access vary among jurisdictions and product characteristics. In the United States, a federal court vacated USDA-APHIS’s 2020 SECURE Rule in December 2024, so its former exemptions should not be assumed to remain universally applicable [113]. Japan has genome-editing notification or prior-information procedures according to the genetic characteristics of the organism [114], while Argentina uses case-by-case prior consultation under Resolution 21/2021 [115]. In England, the precision-breeding framework requires applicable release or marketing procedures, with separate food and feed authorization requirements where relevant [116]. The absence of integrated foreign DNA does not alone guarantee exemption or product approval. Breeding programs should account for intended markets, documentation, seed systems and licensing as well as biological performance. Economic and operational comparisons should include conventional crossing, marker-assisted selection and targeted editing. Where a favorable allele is already available in germplasm, conventional methods may be faster or cheaper; precision editing is attractive when a defined desired allele is absent or linkage drag is problematic. Crop-specific cost per transformation event, number of independent lines required for elite selection and time to cultivar release have not been consistently reported for cucurbits; cross-crop economic analyses should not be presented as such measurements [117]. A realistic timeline includes causal-target validation, delivery and regeneration, molecular confirmation, stable allele recovery, genetic-background development, environmental trials, seed production and regulatory assessment. Agronomic validation should be distinguished from molecular and developmental proof of concept. The *CsHLS1*-edited cucumber line offers a production-relevant example rather than a laboratory phenotype alone. In the study cited as [6], the edited line maintained a total yield comparable to the control under normal planting density; the reported approximately 1.67% higher per-plant yield was not statistically significant. Under one higher-density treatment (458 plants per 100 m^2^), the reported early-stage average fruit yield per plant was approximately 13.4% higher than the control, whereas this advantage was not maintained at 500 plants per 100 m^2^ [6]. These observations support a density-dependent agronomic response, but do not establish a consistent full-season yield advantage across high-density treatments, multi-location stability or commercial cultivar release. Thus, the evidence should be described as cultivation-condition evaluation, not as documented commercial deployment.

Economic and Breeding-Timeline Considerations. The relative merits of genome editing, conventional hybridization and marker-assisted selection (MAS) depend on the causal knowledge of a trait, the availability of useful natural alleles and the genetic background targeted for improvement. When a favorable allele already occurs in compatible germplasm and can be followed by a reliable marker, crossing and MAS may be faster or less expensive than establishing a new transformation and regeneration system. Editing may instead be attractive when a desirable sequence-defined allele is absent from accessible germplasm or when it can be introduced directly into an elite line without linkage drag. However, the full path from target discovery to a cultivar includes construct or reagent preparation, regeneration of independent events, genotyping, inheritance and transgene-status testing where relevant, background recovery, multi-season agronomic trials and seed multiplication; a high editing percentage does not measure time to cultivar release [20,23,25,26,31]. The cost per attempted transformation is also distinct from the cost per independent heritable edited event and the cost per elite, agronomically acceptable line. The studies reviewed here do not provide a consistently defined cucurbit-wide cost per transformation event, the number of events required to deliver one elite line, or a directly comparable editing-versus-MAS cultivar-development timeline. Accordingly, numerical cost savings or universal time advantages cannot be inferred from the available citations. Future reports should separately document laboratory and regeneration inputs, failed events, number of independent confirmed lines, generations and backcrosses required, field-evaluation costs and the ultimate cultivar-development outcome. Genome editing should therefore be viewed as a complementary breeding tool whose economic value must be evaluated case by case, rather than assumed to exceed conventional methods.

## 9. Future Directions for Precision Genome Editing and Breeding in Cucurbits

Future progress in cucurbit genome editing will depend on a transition from predominantly loss-of-function mutagenesis toward precision engineering of allelic variation. Base editing provides a route to introduce defined nucleotide substitutions without generating double-strand breaks and could be particularly valuable for recreating or mimicking sequence-defined alleles underlying flowering and GA- or BR-associated architectural variation [65,72,79]. Prime editing further expands the editable sequence space by enabling targeted substitutions, insertions, and deletions; its successful application in rice and wheat demonstrates the feasibility of this approach in crop species [34], although efficient implementation in cucurbits remains to be established. Beyond coding sequences, cis-regulatory regions represent especially attractive targets for developmental regulators whose complete disruption may cause strong pleiotropic effects. Editing promoters, untranslated regions, or enhancers could generate graded allelic series and thereby enable quantitative tuning of gene activity. The *YABBY1* 5′UTR provides a compelling proof of concept in cucurbits, in which engineered variation in a conserved regulatory region produced dose-dependent changes in stem length and enabled customization of plant architecture [5]. Thus, the future value of precision editing will lie not simply in increasing editing efficiency, but in generating alleles of appropriate functional strength for breeding.

A complementary priority is to move from single-locus manipulation toward rational engineering of allele combinations that coordinate earliness, reproductive development, and plant architecture. Multiplex editing should therefore be guided by developmental relationships among traits rather than by the number of loci that can technically be modified. Multi-sgRNA CRISPR architectures developed in melon and watermelon, including systems capable of tandem sgRNA assembly and dual-target editing, provide an initial technical basis for such strategies [31]. However, useful ideotypes will require balanced phenotypic combinations rather than maximal perturbation of individual pathways. Earlier flowering or enhanced female-flower formation will not necessarily translate into earlier harvest if fruit set or subsequent fruit development becomes limiting, while reduced internode length or branching must be evaluated in the context of canopy function, yield, and environmental adaptation. This need for integrated trait design is underscored by the *CsTIE1*–*CsAGL16* module, which links lateral branch development with drought responses in cucumber [94], as well as by environmental regulatory pathways in which light signaling modulates axillary bud outgrowth [96] and elevated temperature promotes hypocotyl elongation through *CsPIF4*-dependent auxin regulation [97]. Future multiplex-editing studies should therefore prioritize defined allele combinations and evaluate their effects at the whole-plant level under production-relevant environments rather than using molecular editing efficiency as the primary endpoint.

An additional consideration when designing multiplex-editing strategies is the distinction between functional redundancy and functional divergence among members of the same gene family (Supplementary Table S3). Several gene families in cucurbits—including the PEBP family (which includes *FT* and *TFL1*), the *WIP* and *ERECTA* families, growth-regulating factors (*GRFs*), ethylene receptors, and ACS/ACO enzymes—contain multiple members; however, the presence of multiple members within a family does not, by itself, establish that paralogs compensate for one another [7,10,24,37,42,45,46]. Differences in expression domains, developmental roles, or regulatory interactions may explain why disrupting one member produces a strong phenotype, whereas the single-gene knockout of another member has little or no detectable effect. A weak or absent phenotype in a single-mutant line may also reflect incomplete editing, genetic background, or environmental conditions, rather than true functional redundancy. Before targeting multiple paralogs simultaneously, their sequences, expression patterns, and individual loss-of-function phenotypes should therefore be compared. Targeted double or higher-order mutants can then be generated to test whether these genes have compensatory functions. Such combinations should be evaluated for their effects on floral development, stress responses, fruit set, and marketable yield, because the simultaneous disruption of developmental regulators may exacerbate pleiotropic effects. Multi-sgRNA CRISPR systems developed in melon and watermelon provide a technical starting point for testing such combinations [31]; however, technical feasibility alone does not demonstrate that multiplexing paralogs will improve agronomic performance.

Evidence from other crop families provides useful guidance for prioritizing flowering and architecture targets in cucurbits, although the reported phenotypes should not be assumed to transfer directly across species. In rice, *Gn1a* and *OsCKX2* designate the same cytokinin oxidase locus: natural allelic variation affects cytokinin accumulation in reproductive meristems and grain number [118]. The rice *SMALL PANICLE* (*SPA*) locus, studied together with *LAX PANICLE,* provides separate genetic evidence that axillary meristem formation contributes to inflorescence branching [119]. These examples support examining developmental processes underlying reproductive-organ initiation and branching in cucurbits, rather than treating increased organ number as a guaranteed yield benefit. In tomato, editing the flowering repressor *SP5G* accelerated flowering and early yield in the tested genetic backgrounds [120], while a subsequent study combined *SP5G* with *SELF PRUNING* (*SP*), which affects growth termination, and *SlER*, a regulator of internode length, to develop compact, early-yielding plants [121]. This offers a particularly relevant example of combining distinct developmental targets rather than maximizing the effect of a single mutation. In soybean, *Dt1* (*GmTFL1b*) determines stem growth habit and the fate of the shoot apex after floral induction [122], whereas editing Pdh1 improves resistance to pod shattering without establishing a role in flowering or compact growth [123]. These contrasting examples highlight the need to match each editing target to a clearly defined breeding endpoint. Finally, barley *Ppd-H1* and wheat *Ppd-D1* illustrate the importance of photoperiod-response alleles in flowering adaptation [124,125], while barley *Vrs1* demonstrates that a defined developmental regulator can alter spikelet fertility and inflorescence form [126]. For cucurbits, the practical lesson is therefore to select causally supported loci, test modest or combinatorial allelic changes where appropriate, and evaluate flowering time, plant architecture, fruit set, harvest-related traits, and environmental responses together in relevant genetic backgrounds. Such cross-family evidence can guide target selection and experimental design, but cucurbit-specific validation remains essential.

Realizing these designs in breeding germplasm will require parallel advances in editing delivery, regeneration, and target discovery. Current cucurbit transformation and regeneration systems remain strongly genotype dependent, making the development of transgene-free and tissue-culture-independent or -reduced approaches particularly important. In melon, in planta delivery of CRISPR/Cas9 ribonucleoproteins by particle bombardment has enabled heritable, DNA-free editing while bypassing conventional cell-culture regeneration [36]. Developmental regulators have also improved transformation or regeneration efficiency in watermelon and melon [25,26], providing an alternative strategy for recalcitrant genotypes. Further work should focus on increasing germline editing frequencies, extending reproducibility across elite germplasm, and developing transient or removable regeneration-enhancing systems that minimize unintended developmental effects. At the same time, target discovery should increasingly integrate genetic diversity, fine mapping, pangenomic information, and multi-omics datasets. Wild relatives, landraces, mutant populations, and breeding collections represent important sources of functional variation, while resources such as the cucumber EMS mutant library facilitate forward-genetic identification of genes affecting plant architecture and other agronomic traits [127]. Robust QTLs for flowering, sex expression, branching, internode length, and plant height should ultimately be resolved to causal genes or regulatory variants before being prioritized for editing. Integration of these resources with transcriptomic, epigenomic, proteomic, and high-throughput phenotyping data should improve target prioritization and help identify loci with lower pleiotropic risk.

**Figure 2:**
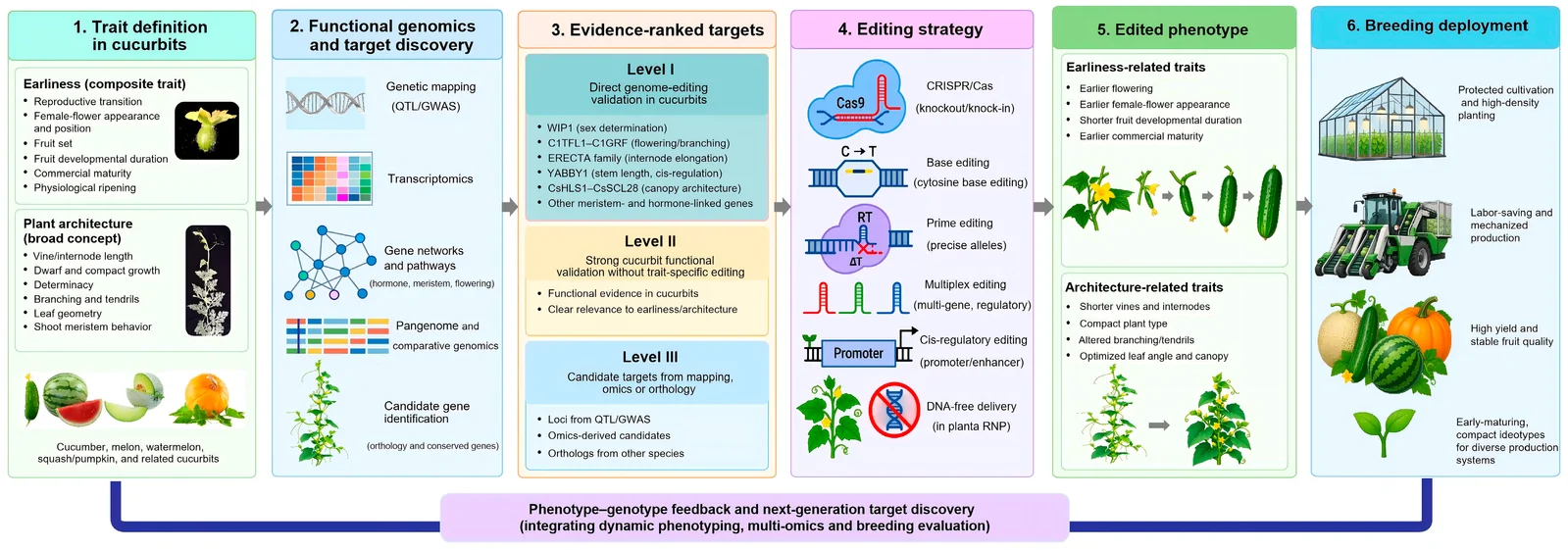
Evidence-guided workflow from trait definition and target validation to allele design, edited-line characterization, production-relevant evaluation and breeding deployment. This diagram describes a proposed framework, not a sequence experimentally completed for any single cucurbit line.

In the longer term, precision allele design and multiplex editing could extend cucurbit improvement from individual-gene modification to developmental-network engineering and, potentially, de novo domestication. Editing multiple domestication-related loci has already enabled rapid modification of plant architecture, fruit size, fruit number, and nutritional traits in wild tomato [128], providing a conceptual framework for exploiting useful diversity in wild cucurbit germplasm while modifying traits that constrain cultivation. For cucurbits, such strategies could seek to retain disease-resistance, stress-adaptation, or quality-associated variation while reshaping reproductive timing, sex expression, stem growth, and branching. The breeding objective should therefore shift toward coordinated optimization of flowering, female-flower formation, fruit set, internode length, branching, canopy organization, yield, quality, and stress adaptation through quantitatively designed allele combinations. Over the next five to ten years, key priorities will include establishing robust editing platforms for elite cucumber, melon, watermelon, and Cucurbita germplasm; converting reproducible QTLs into causal editable variants; generating regulatory and weak-effect allelic series for dosage-sensitive genes; validating defined multiplex combinations under production conditions; and transferring superior allele combinations into breeding pipelines. The development of watermelon germplasm combining dwarf and branchless mutations already demonstrates that favorable architecture alleles can be successfully pyramided [100]. Genome editing should make such combinations faster to construct, more precisely tunable, and more readily testable across genetic backgrounds and environments. An evidence-guided workflow integrating trait definition, functional genomics, target prioritization, precision allele design, phenotypic evaluation, and breeding deployment is proposed to guide the translation of genome-editing advances into practical cucurbit improvement (Figure 2).

## 10. Conclusions

Genome editing has strengthened the functional validation of genes controlling flowering, sex expression, internode length, branching and canopy architecture in cucurbits. However, direct editing, functional evidence and candidate mapping differ in evidentiary strength, and none alone establishes commercial breeding value. The evidence framework used here therefore separates causal knowledge, technical editability and agronomic deployment.

A central unresolved question is whether the interval from fruit set to crop-specific commercial harvest can be shortened without sacrificing fruit size, quality or yield. Future studies should define and measure separate endpoints for flowering, female-flower formation, fruit set, developmental duration, marketable harvest and early production. Genes influencing ripening or fruit size require direct testing before being designated early-harvest targets.

An additional priority is to obtain quantitatively tuned alleles that avoid the severe pleiotropy of strong loss-of-function mutations. *ERECTA*-related knockouts, YABBY1 cis-regulatory alleles and flowering–branching modules illustrate both opportunity and biological risk [5,23,24]. Partial-loss-of-function, regulatory editing and carefully evaluated multi-locus combinations should be judged by whole-plant fertility, source–sink relations and marketable productivity, not by the strength of dwarfism or early flowering alone.

Trait definition establishes breeding objectives for early-maturing and compact ideotypes; functional genomics identifies candidate genes; evidence ranking prioritizes editing targets; tailored genome-editing strategies generate desired alleles; edited phenotypes are evaluated for earliness, architecture, and agronomic performance; and superior traits are deployed in breeding programs. Phenotype-genotype feedback further refines target discovery and supports iterative quantitative ideotype design.

## Figures and Tables

**Table 1:** Genome-editing platforms and delivery systems in major cucurbit crops.

Crop(s)	Platform/Delivery	Representative Target or Use	Status	Breeding Relevance	Reference
Cucumber	CRISPR/Cas9; transgene-free by segregation	*CsWIP1*; virus-resistance proof of concept	Established	Stable edited progeny and reproductive-trait engineering	[20,21]
Watermelon	CRISPR/Cas9	Gene knockout; *ClWIP1*	Established	Trait-directed knockout and sex-expression engineering	[19,22]
Cucumber, melon, squash	Optimized Agrobacterium + CRISPR/Cas9	*ERECTA* homologs	Cross-species proof-of-concept	Heritable compact-architecture mutants in three cucurbit crops	[23]
Watermelon	Developmental-regulator-assisted transformation	*AtGRF5*; PDS editing	Emerging	Improves regeneration/editing competence	[25]
Melon	Developmental-regulator-assisted transformation	*AtGRF5/AtPLT5*; *CmPDS*	Emerging	Improves regeneration across genotypes	[26]
Melon	Cytosine base editing	*CmeIF4E*	Proof-of-concept	Defined nucleotide conversion	[32]
Melon, watermelon	Pol II–Csy4 CRISPR/Cas9	Paired targets/large deletions	Emerging	Foundation for multiplex/structural editing	[31]
Melon	DNA-free in planta Cas9 RNP	*CmACO1*	Proof-of-concept	Heritable editing without conventional cell culture	[36]
Cucumber, Melon, pumpkin	Optimized Csy4-PE6d prime editing system; SlEF1α-driven editor expression and Csy4-mediated pegRNA processing	No validated cucurbit earliness/architecture deployment in this evidence set	Experimentally validated	Potential for precise recreation or introduction of favorable alleles	[34]

**Table 2:** Evidence classification and breeding relevance of earliness-related genes and loci in cucurbit crops.

Crop	Gene/Locus	Trait/Pathway	Evidence	Editing/Functional Evidence and Phenotype	Breeding Interpretation/Limitation	Reference
Cucumber	*CsWIP1*	Sex determination; WIP/ethylene network	Level I	CRISPR/Cas9 disruption; strongly increased femaleness/gynoecious phenotype	Female-flower/sex-expression engineering and hybrid seed production; earlier commercial maturity not directly shown	[21]
Watermelon	*ClWIP1*	Sex determination	Level I	CRISPR/Cas9 editing; gynoecious watermelon lines	Female-flower/sex-expression engineering; earlier fruit maturity not directly shown	[22]
Watermelon	*ClTFL1*; *ClGRF8/12*	Flowering–branching; auxin-linked module	Level I	CRISPR/Cas9 knockouts; earlier flowering and reduced lateral branching; *ClTFL1* also affects tendrils	Joint earliness–architecture target; pleiotropy and allele strength require evaluation	[24]
Watermelon	*ClPID*	Auxin-linked lateral-organ development	Level I	CRISPR/Cas9 knockout; earlier flowering, dwarfism, floral-organ defects, and round leaves	Direct flowering evidence, but strong null alleles are not breeder-ready because of pleiotropy	[17]
Watermelon	*ClAP1*	Floral initiation/floral meristem identity	Level I (flowering function)	CRISPR/Cas9 knockout inhibits floral initiation	Direct editing validates floral function, but loss of floral initiation is not an earliness breeding phenotype	[17]
Cucumber	*ACS1G*	Ethylene biosynthesis; gynoecy/female development	Level I	CRISPR/Cas9 mutagenesis combined with EMS and transformation analyses established *ACS1G* as the F-locus gene responsible for gynoecy	Sex-expression engineering; increased female flowering does not by itself demonstrate earlier commercial maturity	[42]
Cucumber	*CsARF3*	Auxin–ethylene crosstalk; carpel development and sex determination	Level I	Direct deletion produces androecy; overexpression increases the number of female flowers	Strong sex-expression target; allele direction and pleiotropic effects require careful testing	[49]
Melon	*CmACO1*	Ethylene biosynthesis; fruit ripening/shelf life	Level I (fruit development)	DNA-free in planta CRISPR/Cas9 RNP editing; reduced ethylene production and extended shelf life	Important DNA-free editing case, but delayed ripening/longer shelf life is not evidence of earlier commercial maturity	[36]
Cucumber	*CsTFL1/CsTFL1d*	Meristem fate; determinacy; temperature/photoperiod response	Level II	Natural/deletion alleles, complementation, expression, knockdown, and interaction evidence; focal earliness genome editing not established	Potential regulatory or weak-allele targets; environmental responsiveness should be tested	[7,37]
Cucumber	*CsPHYB*	Light signaling; flowering time	Level II	Natural 5.5 kb LTR-retrotransposon insertion in *CsPHYB* is associated with long hypocotyls and early flowering	Candidate for favorable-allele recreation or regulatory editing; test stability across light and temperature environments	[11]
Cucumber	*CsLFY/CsWUS*	Shoot-meristem maintenance; flower development	Level II	*CsLFY* RNAi causes defective shoot development; *CsLFY* interacts with *CsWUS* and promotes flower-development genes	Mechanistically relevant to reproductive development, but focal earliness genome editing is not established	[38]
Cucumber	*CsCLV2*	Shoot-apical-meristem maintenance; determinacy	Level II (for earliness)	CRISPR-Cas9 loss-of-function recapitulates dwarf/determinate growth through premature SAM termination; no direct earlier-flowering or maturity phenotype established	Directly editable architecture regulator, but evidence does not establish an earliness allele; strong pleiotropy risk	[39]
Watermelon	*CitACS4*	Ethylene biosynthesis; monoecy/andromonoecy	Level II	A missense allele cosegregates with andromonoecy and reduced ethylene production in pistillate floral buds; no focal genome-editing validation	Sex-expression target; the reported allele does not establish earlier female flowering or earlier maturity	[43]
*C. pepo*	*CpETR1A*/*CpETR1B*/*CpETR2B*	Ethylene perception; female-flower initiation/transition	Level II	EMS-derived ethylene-receptor mutations alter female-flower initiation, sex phenotype, and female flowering transition	Potential receptor-level tuning; allele dosage and broad ethylene-response pleiotropy require caution	[45,46]
*C. pepo*	*CpACO1A*	Ethylene biosynthesis; sex determination and female-flower development	Level II	EMS missense mutant reduces ACO activity and ethylene, converts female flowers toward bisexual/hermaphrodite flowers, and delays female-flower opening	Functional sex-development target; no focal genome-editing validation and no evidence of earlier commercial maturity	[47]
*C. pepo*	*CpMYB62*	Transcriptional control of female-flower determination	Level II	EMS loss-of-function mutation blocks the female floral transition and produces androecy; functional and genetic analyses place *CpMYB62* upstream in the sex-determination network	Potential regulatory target for female flowering; no focal genome-editing validation	[48]
Cucumber	*CsERF31*	Ethylene-response feedback; female-flower development	Level II	RNAi knockdown and ectopic-expression analyses support a positive-feedback role in female flower development	Potential organ- or stage-specific tuning target; no focal genome-editing validation	[50]
*C. pepo*	*CpCUC2B*	Carpel arrest; floral boundary specification	Level II	An EMS-derived miR164-resistant gain-of-function mutation alters carpel arrest, floral organs, and sex expression	Mechanistically relevant to sex determination, but strong pleiotropy and lack of focal genome-editing validation limit breeding readiness	[55]
*C. pepo*	*CpLOX3A*	Jasmonate biosynthesis; flower opening and early fruit development	Level II	EMS loss-of-function mutant suppresses flower opening and promotes parthenocarpic fruit development; phenotype is rescued by methyl jasmonate	Supports hormone control of anthesis and early fruit development, but does not demonstrate earlier commercial maturity	[52]
Cucumber	SF1/ACS2	Ethylene dosage; fruit cell division and elongation	Level II	Mutant and biochemical evidence shows SF1 controls ACS2 stability and ethylene dosage, affecting cell division and fruit elongation	Relevant to fruit developmental biology, but no direct evidence that editing shortens anthesis-to-commercial-harvest duration	[60]
Watermelon	Partial-andromonoecy (pa) locus	Sex expression; partial andromonoecy	Level III	BSA-seq, fine mapping, and GWAS identify a chromosome-1 locus; the causal gene remains unresolved	Resolve the causal gene/variant and effect direction before genome editing	[44]
Zucchini	Early female-flowering QTLs	Days/node to early female flowering	Level III	QTL mapping identifies loci associated with early female flowering	Fine-map to the causal gene/allele and validate effects before editing	[4]
Citron watermelon	Flowering-time QTLs	Flowering time and fruit yield	Level III	QTL evidence for flowering-time and yield-related variation	Resolve causal variants and test environmental stability before editing	[3]
Bitter gourd	Earliness QTLs	Earliness, fruit, and seed-related traits	Level III	High-density GBS-based QTL mapping identifies loci for earliness-related traits	Causal-gene resolution and validation are required before editing	[2]
Cucumber	Flower- and fruit-related QTLs	First flower node; first female-flower node; female-flower ratio; fruit traits	Level III	RIL-based QTL mapping identifies multiple loci for flower and fruit traits	Useful for candidate discovery, but mapped intervals require causal resolution before editing	[58]
Luffa	Maturity- and flowering-related QTLs	First male/female flower node; first fruit node; daily flowering time	Level III	High-resolution QTL mapping identifies multiple maturity-related loci and candidate genes	Fine-map and functionally validate causal genes/alleles before genome editing	[64]

Note: Level I indicates direct genome-editing evidence in cucurbits; Level II indicates functional evidence without direct editing validation of the target trait; and Level III indicates candidate loci supported primarily by genetic mapping or association analyses. The qualifiers “flowering function” and “fruit development” specify the phenotypes validated by editing, rather than demonstrated improvements in earliness.

**Table 3:** Evidence classification and breeding relevance of plant-architecture genes and loci in cucurbit crops.

Crop	Gene/Module	Architecture Process	Evidence	Editing/Phenotype	Breeding Value/Limitation	Reference
Cucumber, melon, squash	*ERECTA* homologs	Internode elongation	Level I	CRISPR knockout; short internodes and compact growth	Cross-species compactness; allele strength should be tuned	[23]
Cucumber	*CsERECTA*	GA-linked stem elongation	Level I	CRISPR knockout; dwarfing and reduced GA	Mechanistically supported compactness target; avoid excessive reduction	[67]
Pumpkin, cucumber, watermelon	*YABBY1* 5′UTR	Stem-length regulation	Level I	CRISPR cis-regulatory deletions; quantitative stem shortening	Strong model for tunable regulatory editing	[5]
Cucumber	*CsHLS1* (*CsHLS1*–*CsSCL28* module)	Petiole angle; leaf size; canopy width	Level I	*CsHLS1* CRISPR knockout; reduced petiole angle, leaf size, and plant width	Density-oriented canopy target; full-season high-density yield still needs validation	[6]
Cucumber	*CsCLV2*	Meristem maintenance/determinacy	Level I	Edited loss-of-function; dwarf, determinate growth	Compact/determinate architecture; premature meristem termination is a major risk	[39]
Watermelon, cucumber	*ClDUF21*/*CsDUF21*	BR-linked internode growth	Level I	CRISPR; dwarfing/shortened internodes	Conserved compactness target; severe dwarfing risk	[80]
Watermelon	*ClTFL1*; *ClGRF8/12*	Branching + flowering	Level I	CRISPR; fewer branches and earlier flowering	Joint earliness–architecture target; pleiotropy	[24]
Watermelon	*ClPID*/*ClLs*/*ClTEN*	Lateral-organ development	Level I	Direct editing; dwarf/early-flowering or lateral-organ defects depending on gene	Strong functional evidence; substantial pleiotropic risk	[17]
Cucumber	*CsIAGLU*	Petiole angle; auxin homeostasis	Level I	CRISPR knockout; increased petiole angle	Knockout direction is not ideal for compact canopy; favor small-angle alleles/increased activity	[102]
Cucumber	*CsIREH1*	DELLA stability; plant height	Level I	CRISPR disruption; strong growth retardation/reduced plant height	Direct architecture target; strong phenotype suggests need for weaker alleles	[69]
Cucumber	*CsTIE1* (*CsTIE1*–*CsAGL16* module)	Lateral branching and drought response	Level I	CRISPR knockout; reduced branching and drought tolerance	Reduced branching; drought tolerance trade-off	[94]
Cucumber	*CsAGL16*-*CsGRF1* module	Axillary-bud outgrowth via ABA catabolism	Level I	CRISPR knockout; *Csagl16* mutant suppresses and Csgrf1 mutant enhances branching	Tunable branching; ABA homeostasis requires balancing	[95]
*Cucumber*	*CsPHYB* *–CsPIF4–CsBRC1 module*	Light-regulated ABA biosynthesis and branching	Level I	Gene disruption; *CsPHYB* suppresses, whereas CsPIF4/CsNCED3 loss enhances bud outgrowth	Light-responsive branching control; pleiotropic effects need evaluation	[96]
Watermelon	*ClNAC100*	GA-linked plant height; fruit size	Level I	CRISPR/Cas9 knockout reduces plant height and fruit size	Compactness target with a strong fruit-size trade-off	[87]
Cucumber	*CsRAX1/2/5*	Leaf size; stem growth; fruiting ability	Level I	CRISPR single/multiple mutants alter leaf/stem size and fruiting ability	Potential canopy/yield target; allele direction and strength require optimization	[106]
Watermelon	*ClLMI1*	Lobed-leaf development; auxin distribution	Level I	CRISPR/Cas9 knockout validates lobed-leaf function	Leaf-shape target; production-system value still requires evaluation	[107]
Cucumber	*CsTFL1*	Meristem fate/determinacy	Level II	Natural/functionally validated alleles; no focal architecture editing established here	Regulatory or weaker alleles may be preferable	[37,65]
Watermelon	Clbl (*TFL1* protein)	Branch number	Level II	Natural/functionally validated branchless allele	Branch load must be matched to fruiting habit	[66]
Cucumber	*CsBRC1*–*CsPIN3*	Axillary-bud outgrowth	Level II	RNAi/transgenic and biochemical functional evidence; no focal CRISPR editing	Reduce pruning while retaining productive lateral shoots	[93]
Cucumber	*CsABCB19*	Leaf/petiole architecture	Level II	EMS mutant, mapping, and functional validation	Canopy-angle target; direct focal editing not established	[101]
Cucumber	*CsKAO*	GA biosynthesis; plant height	Level II	EMS mutant; causal mapping; GA3 rescue	Favorable allele recreation possible; severe GA deficiency undesirable	[68]
Cucumber	*CsDELLA1*	GA signaling/growth	Level II	Functional characterization; no focal genome editing established	Potential GA-response tuning target	[70]
Cucumber	*CsDET2*/*CsDWF5*/*CsDWF7*	BR biosynthesis	Level II	Dwarf/super-compact mutants	Moderate allele tuning preferable to severe BR deficiency	[77,78,79]
Watermelon	*Cldf*/GA3ox	GA biosynthesis	Level II	Natural/induced dwarf alleles	Moderate compactness; vigor trade-off	[71,72]
Squash	*CpDw*/*CmaGA2ox2*	GA metabolism	Level II	Dwarf alleles	Potential favorable-allele recreation or tuning	[73,74]
Sponge gourd	*LacDWARF1*	GA-related stem growth	Level II	Mutant functional evidence; GA-deficient dwarfism	Potential compactness target; avoid excessive dwarfism	[75]
Watermelon	*Cldw-1*/ABC-transporter dwarf loci	Auxin transport; plant height	Level II	Natural mutant/functional evidence	Compactness target; vigor and reproductive performance require evaluation	[81,82]
Melon	*CmSi*	*ERECTA*-like/auxin signaling	Level II	Functional short-internode allele	Candidate for calibrated compactness	[83]
Cucumber	*CsSMR1*	Cell-cycle control; internode growth/determinacy	Level II	Natural genomic deletion; causal mapping; dwarf determinate phenotype	Strong architecture effect; determinacy and pleiotropy must be managed	[84]
Melon	*CmSVPc*	Lateral-branch development	Level II	RNAi in melon and overexpression evidence	Branching target; also affects internodes and other organs	[98]
Cucumber	*CsWOX1*	Leaf size/vein patterning	Level II	Mutant, complementation, and overexpression evidence	Leaf-size target; developmental effects require evaluation	[103]
Cucumber	*CsGME*	Leaf size/shape	Level II	Mapped mutant plus VIGS functional validation	Small/wrinkled-leaf phenotype may limit direct breeding use	[105]
Watermelon	*Cldw-1* + Clbl	Plant height + branching	Level II	Pyramided natural architecture alleles; dwarf-branchless germplasm	Demonstrates feasibility of architecture-allele stacking before multiplex editing	[100]
Cucumber	*CsCYP85A1*/Scp-1 region	BR-linked compactness	Level III	Putative candidate from genetic mapping	Causal relationship should be resolved before editing	[76]
Cucumber	cp locus/CKX homolog	Compact/dwarf architecture	Level III	Fine-mapped region; CKX homolog proposed as candidate	Causal gene/variant requires functional validation	[85]
Cucumber	*CsCLAVATA1*	Dwarf architecture	Level III	Putative candidate associated with dwarf mutation	Functional validation required before editing	[86]
Watermelon	Plant-height/dwarf loci	Plant height	Level III	Fine-mapping/BSA candidate loci	Resolve causal genes and alleles before editing	[88,89]
Melon	Short-internode region	Internode length	Level III	BSA-associated genomic region	Fine-map and validate causal allele	[90]
Winter squash	Internode-length loci	Internode length	Level III	Quantitative genetic evidence	Resolve major genes and polygenic background before editing	[91]
Pumpkin/*C. pepo*	Dwarf-vine/plant-height QTLs	Vine/plant height	Level III	QTL/mapping evidence	Causal-allele resolution required	[92,109]
Melon	Branch-number QTLs	Axillary branching	Level III	QTL candidates	Fine-map before editing	[99]
Cucumber	*LITTLELEAF*-associated networks	Leaf/organ size	Level III	Transcriptomic network evidence	Identify causal regulators before editing	[104]
Melon	Leaf stalk–stem angle pathways	Leaf/canopy angle	Level III	Comparative transcriptome evidence	Resolve causal genes before editing	[108]

Note: Level I, direct genome editing in a cucurbit species with a reported relevant architectural phenotype; Level II, strong cucurbit-specific functional evidence without direct genome-editing validation of the focal architecture trait; Level III, support mainly from mapping, QTL/BSA, transcriptomics, or unresolved candidate-gene evidence. Quantitative comparisons require matched genotype, growth environment and endpoint; NR means not reported in the cited source, not merely not extracted.

## Data Availability

No new data were created or analyzed in this study. Data sharing is not applicable to this article.

## Supplementary Materials

The following supporting information can be downloaded at: https://www.mdpi.com/article/10.3390/ijms27219697/s1.
