# Supplementary material for: Genome Editing for Earliness and Plant Architecture in Cucurbit Crops: Recent Advances and Future Perspectives
Source: Int J Mol Sci. 2026 Oct 29;27(21):9697. doi: 10.3390/ijms27219697 (PMC13762802; doi:10.3390/ijms27219697)
Supplement: Supplementary file 1 [file ijms-27-09697-s001.zip › ijms-4601846-supplementary.pdf]

Supplementary Table S1. Chromosome and Genome Context

Species	Chromosome number	Current ploidy	Assembly/genomic context	Ref.
Cucumis sativus	2n = 14	Diploid	Original cucumber assembly; version-specific size	[27]
Cucumis melo	2n = 24	Diploid	DHL92 assembly: approximately 375 Mb; genome estimate differs	[28]
Citrullus lanatus	2n = 22	Diploid	Reference genome; accession-dependent estimates	[29]
Cucurbita pepo	2n = 40	Diploid cytotype; ancestral WGD	Zucchini assembly: approximately 263 Mb	[30]

Assembly length, estimated genome size, cytological ploidy and ancestral whole-genome duplication are distinct measurements. Values for additional Cucurbita, Luffa and Momordica species require accession- and version-specific verification.

Supplementary Table S2. Reported Editing and Phenotypic Evidence

Crop	Platform/target	Genetic background	Reported outcome	Evidence limitation	Ref.
Melon	CmER CRISPR/Cas9	m1	Reduces plant height by approximately 40%, producing compact dwarf plants in edited material	3.95%transformation efficiency;23.47 – 40.69% editing rate;Chimerism rate NR; Transgene-free progeny NR	[23]
Cucumber	CsER CRISPR/Cas9	Cu2	Shortens internodes by 34%, resulting in more compact cucumber plants in edited material	5.18%transformation efficiency;31.95 – 36.49% editing rate;Chimerism rate NR; Transgene-free progeny NR	[23]
Squash	CmoER1,Cmo	Jingxinzhen	CmoER double	3.56%transformat	[23]

	ER2 CRISPR/Cas9	No. 4	knockout shortens internodes by 60%, producing pronounced dwarf plant architecture.	ion efficiency;18.94 – 25.78%CmoER1 / 22.66 – 39.49%CmoER2 editing rate;Chimerism rate NR; Transgene-free progeny NR	
Watermelon	AtGRF5-assisted transformation/ ClPDS	WW150; 83166	24.73% (WW150) and 20.72%(83166) transformation efficiency	27.27%(WW150) editing rate;Chimerism rate NR; Transgene-free progeny NR;11 T0 transgenic plants, including 3 edited plants	[25]
Melon	Pol II–Csy4 / CmPDS	Tested melon materials Vedrantasis	42.82% edited regenerated plants	Chimerism / transgene-free progeny not comparable here	[31]
Watermelon	Pol II–Csy4 / ClPDS	Tested watermelon materials D66	41.48% edited regenerated plants	Not a stable-line recovery percentage	[31]
Melon	Pol II–tRNA / CmPDS	Tested melon materials	25.20% edited regenerated plants	Not a transformation percentage	[31]
Cucumber	CsTIE1 CRISPR/Cas9	R1461	Fewer and shorter lateral branches, reduced drought tolerance, and increased wilting in edited material	Nine independent stable edited lines reported; Chimerism rate NR; Transgene-free progeny NR; early yield NR	[94]
Cucumber	CsAGL16 CRISPR/Cas9	WI1983M	Suppresses axillary bud outgrowth and reduces drought	Two homozygous edited lines; Chimerism rate NR;	[95]

			tolerance.	Transgene-free progeny NR; early yield NR	
Cucumber	CsCYP707A4 CRISPR/Cas9	WI1983M	Increases bud ABA accumulation, suppressing lateral branch outgrowth.	Two homozygous edited lines; Chimerism rate NR; Transgene-free progeny NR; early yield NR	[95]
Cucumber	CsGRF1 CRISPR/Cas9	WI1983M	Branching increased in edited material	No universal cross-study percent or yield claim	[95]
Cucumber	CsPIF4 functional disruption	transformation recipient unverified	Reduces bud ABA accumulation and promotes lateral branch outgrowth.	Different study confirms CRISPR for hypocotyl trait	[96,97]
Cucumber	CsHLS1 CRISPR/Cas9	KT(North China type)	Reduces leaf petiole angles and leaf size, producing compact architecture.	two homozygous edited lines.Editing frequency NR;Chimerism rate;Proportion of transgene-free progeny NR	[6]
Cucumber	CsSCL28 CRISPR/Cas9	649 inbred line	Reduces leaf petiole angles and leaf size, producing compact architecture.	two homozygous edited lines.Editing frequency NR;Chimerism rate;Proportion of transgene-free progeny NR	[6]

NR = not reported in the available citation-supported information

Supplementary Table S3. Major Gene Families and Regulatory Modules

Gene family or module	Representative cucurbit genes	Functional interpretation	Original refs
PEBP / FT–TFL1	CsTFL1; CsTFL1d; ClTFL1; Clbl	Flowering, determinacy, branching	[7,10,24,37,65,66]
WIP zinc-finger	CsWIP1; ClWIP1	Sex expression and female-flower development	[21,22,40]
NOT-associated	CsNOT2a	CsTFL1-associated shoot-meristem regulation	[37]
PHYB/photoreceptor	CsPHYB	Light-linked flowering and branching	[11,96]
CLAVATA	CsCLV2; CsCLAVATA1	Shoot-meristem maintenance and stature	[39,86]
WUS / WOX	CsWUS; CsWOX1	Meristem maintenance; leaf development	[38,103]
LFY	CsLFY	Shoot meristem and floral development	[38]
ACS	ACS1G; CitACS4; ACS2	Ethylene biosynthesis; sex and fruit phenotypes	[42,43,60]
ACO	CmACO1; CpACO1A	Ethylene biosynthesis and fruit ripening / sex	[36,47]
ETR receptors	CpETR1A; CpETR1B; CpETR2B	Ethylene perception; female flower initiation	[45,46]
MYB	CpMYB62	Female-flower transition	[48]
ERF	CsERF31	Female-flower ethylene-response feedback	[50]
ARF	CsARF3	Auxin-associated carpel and sex development	[49]
Growth-regulating factor (GRF)	ClGRF8; ClGRF12	Flowering–branching module	[24]
GENERAL REGULATORY FACTOR / 14-3-3	CsGRF1	Interaction with CsAGL16; ABA catabolism and branching	[95]
ERECTA receptor kinases	CsERECTA; CmSi; ERECTA homologs	Stem elongation and compactness	[23,67,83]

YABBY	YABBY1	Quantitative stem length via 5′UTR	[5]
DELLA / GA-related	CsDELLA1; CsIREH1; CsKAO; CmaGA2ox2	Gibberellin-mediated growth	[68,69,70,74]
BR-related metabolism	CsDET2; CsDWF5; CsDWF7; ClDWF1	BR-related compact growth	[77,78,79,80]
DUF21	ClDUF21; CsDUF21	Internode growth	[80]
BRC1 / TCP	CsBRC1; tendril-associated TCP	Branching and lateral-organ development	[18,93,96]
TIE–AGL	CsTIE1; CsAGL16	ABA catabolism; branching and drought response	[94,95]
PIF	CsPIF4	Light / ABA-regulated branching; thermoresponses	[96,97]
PIN	CmPIN2; CsPIN3	Auxin transport	[83,93]
ABCB	CsABCB19; Cldw-1	Auxin transport; petiole and stature	[81,101]
HLS–SCL	CsHLS1; CsSCL28	Petiole angle and canopy width	[6]
RAX	CsRAX1/2/5	Leaf, stem and fruiting phenotypes	[106]
CUC	CpCUC2B	Floral boundary and carpel arrest	[55]
LOX	CpLOX3A	Jasmonate, flower opening and fruit development	[52]
NAC	ClNAC100	Plant height and fruit size	[87]
LMI / homeodomain	ClLMI1	Leaf lobing	[107]
SMR	CsSMR1	Cell cycle, growth and determinacy	[84]

Note: Functional annotation does not imply that all individual family members have direct genome-editing validation for each associated trait. CsGRF1 is a 14-3-3/general regulatory factor, not a member of the watermelon ClGRF8/ClGRF12 growth-regulating-factor group.
